# Homo-oligomerization of the human adenosine A_2A_ receptor is driven by the intrinsically disordered C-terminus

**DOI:** 10.7554/eLife.66662

**Published:** 2021-07-16

**Authors:** Khanh Dinh Quoc Nguyen, Michael Vigers, Eric Sefah, Susanna Seppälä, Jennifer Paige Hoover, Nicole Star Schonenbach, Blake Mertz, Michelle Ann O'Malley, Songi Han

**Affiliations:** 1 Department of Chemistry and Biochemistry, University of California, Santa BarbaraSanta BarbaraUnited States; 2 Department of Chemical Engineering, University of California, Santa BarbaraSanta BarbaraUnited States; 3 C. Eugene Bennett Department of Chemistry, West Virginia UniversityMorgantownUnited States; Michigan State UniversityUnited States; Weill Cornell MedicineUnited States

**Keywords:** c-terminus, oligomerization, gpcrs, adenosine a2a receptor, depletion interactions, intrinsically disordered regions, *E. coli*, *S. cerevisiae*

## Abstract

G protein-coupled receptors (GPCRs) have long been shown to exist as oligomers with functional properties distinct from those of the monomeric counterparts, but the driving factors of oligomerization remain relatively unexplored. Herein, we focus on the human adenosine A_2A_ receptor (A_2A_R), a model GPCR that forms oligomers both in vitro and in vivo. Combining experimental and computational approaches, we discover that the intrinsically disordered C-terminus of A_2A_R drives receptor homo-oligomerization. The formation of A_2A_R oligomers declines progressively with the shortening of the C-terminus. Multiple interaction types are responsible for A_2A_R oligomerization, including disulfide linkages, hydrogen bonds, electrostatic interactions, and hydrophobic interactions. These interactions are enhanced by depletion interactions, giving rise to a tunable network of bonds that allow A_2A_R oligomers to adopt multiple interfaces. This study uncovers the disordered C-terminus as a prominent driving factor for the oligomerization of a GPCR, offering important insight into the effect of C-terminus modification on receptor oligomerization of A_2A_R and other GPCRs reconstituted in vitro for biophysical studies.

## Introduction

G protein-coupled receptors (GPCRs) have long been studied as monomeric units, but accumulating evidence demonstrates that these receptors can also form homo- and hetero-oligomers with far-reaching functional implications. The properties emerging from these oligomers can be distinct from those of the monomeric protomers in ligand binding (El-Asmar et al., 2005; Casadó-Anguera et al., 2016; Guitart et al., 2014; Yoshioka et al., 2001), G protein coupling (Cristóvão-Ferreira et al., 2013; Cordomí et al., 2015; González-Maeso et al., 2007; Lee et al., 2004; Rashid et al., 2007), downstream signaling (Liu et al., 2016; Hilairet et al., 2003; Rozenfeld and Devi, 2007; Borroto-Escuela et al., 2010), and receptor internalization/desensitization (Ecke et al., 2008; Stanasila et al., 2003; Faklaris et al., 2015). With the vast number of genes identified in the human genome (Takeda et al., 2002), GPCRs are able to form a daunting number of combinations with unprecedented functional consequences. The existence of this intricate network of interactions among GPCRs presents major challenges and opportunities for the development of novel therapeutic approaches (Dorsam and Gutkind, 2007; Farran, 2017; Schonenbach et al., 2015; Ferré et al., 2014; Bräuner-Osborne et al., 2007; George et al., 2002). Hence, it is crucial to identify the driving factors of GPCR oligomerization, such that this process can be more deliberately controlled to facilitate structure-function studies of GPCRs.

GPCR oligomers with multiple interfaces (Song et al., 2020; Ghosh et al., 2014; Periole et al., 2012; Fanelli and Felline, 2011; Liu et al., 2012) can give rise to myriad ways by which these complexes can be formed and their functions modulated. In the crystal structure of the turkey β_1_-adrenergic receptor (β_1_AR), the receptor appears to dimerize via two different interfaces, one formed via TM4/TM5 (transmembrane domains 4/5) and the other via TM1/TM2/H8 (helix 8) contacts (Huang et al., 2013). Similarly, in the crystal structure of the antagonist-bound μ-opioid receptor (μ-OR), the protomers also dimerize via two interfaces; however, only one of them is predicted to induce a steric hindrance that prevents activation of both protomers (Manglik et al., 2012), hinting at interface-specific functional consequences. A recent computational study predicted that the adenosine A_2A_ receptor (A_2A_R) forms homodimers via three different interfaces and that the resulting dimeric architectures can modulate receptor function in different or even opposite ways (Fanelli and Felline, 2011). All the above-mentioned interfaces are symmetric, meaning that the two protomers are in face-to-face orientations, hence forming strictly dimers. Asymmetric interfaces, reported in M_3_ muscarinic receptor (Thorsen et al., 2014), rhodopsin (Fotiadis et al., 2006; Fotiadis et al., 2003; Liang et al., 2003), and opsin (Liang et al., 2003), are in contrast formed with the protomers positioning face-to-back, possibly enabling the association of higher-order oligomers.

Not only do GPCRs adopt multiple oligomeric interfaces, but various studies also suggest that these interfaces may dynamically rearrange to activate receptor function (Xue et al., 2015). According to a recent computational study, A_2A_R oligomers can adopt eight different interfaces that interconvert when the receptor is activated or when there are changes in the local membrane environment (Song et al., 2020). Similarly, a recent study that combined experimental and computational data proposed that neurotensin receptor 1 (NTS_1_R) dimer is formed by ‘rolling’ interfaces that coexist and interconvert when the receptor is activated (Dijkman et al., 2018). Clearly, meaningful functional studies of GPCRs require exploring their dynamic, heterogeneous oligomeric interfaces.

The variable nature of GPCR oligomeric interfaces suggests that protomers of GPCR oligomers may be connected by tunable interactions. In this study, we explore the role of an intrinsically disordered region (IDR) of a model GPCR that could engage in diverse non-covalent interactions, such as electrostatic interactions, hydrogen bonds, or hydrophobic interactions. These non-covalent interactions are readily tunable by external factors, such as pH, salts, and solutes, and further can be entropically enhanced by depletion interactions (Asakura and Oosawa, 1958; Yodh et al., 2001; Marenduzzo et al., 2006), leading to structure formation and assembly (Milles et al., 2018; Wicky et al., 2017; Szasz et al., 2011; Goldenberg and Argyle, 2014; Qin and Zhou, 2013; Cino et al., 2012; Soranno et al., 2014; Zosel et al., 2020). In a system where large protein molecules and small solute particles typically coexist in solution, assembly of the protein molecules causes their excluded volumes to overlap and the solvent volume accessible to the non-protein solutes to increase, raising the entropy of the system. The type and concentration of solutes or ions can also remove water from the hydration shell around the proteins, further enhancing entropy-driven protein-protein association in what is known as the hydrophobic effect (Tanford, 1980; Tanford, 1978; Pratt and Chandler, 1977; van der Vegt et al., 2017). This phenomenon is applied in the precipitation of proteins upon addition of so-called salting-out ions according to the Hofmeister series (Hofmeister, 1888; Hyde et al., 2017; Yang, 2009). The ability of IDRs to readily engage in these non-covalent interactions motivates our focus on the potential role of IDRs in driving GPCR oligomerization.

The cytosolic carboxy (C-)terminus of GPCRs is usually an IDR (Tovo-Rodrigues et al., 2014; Jaakola et al., 2005). Varying in length among different GPCRs, the C-terminus is commonly removed in structural studies of GPCRs to enhance receptor stability and conformational homogeneity. A striking example is A_2A_R, a model GPCR with a particularly long, 122-residue, C-terminus that is truncated in all published structural biology studies (Song et al., 2020; Fanelli and Felline, 2011; García-Nafría et al., 2018; Sun et al., 2017; Lebon et al., 2011; Xu et al., 2011; Doré et al., 2011; Jaakola et al., 2008; Carpenter et al., 2016; Hino et al., 2012). However, evidence is accumulating that such truncations—shown to affect GPCR downstream signaling (Koretz et al., 2021; Navarro et al., 2018a; Jain and McGraw, 2020)—may abolish receptor oligomerization (Schonenbach et al., 2016; Svetlana and Devi, 1997). A study using immunofluorescence has demonstrated that C-terminally truncated A_2A_R does not show protein aggregation or clustering on the cell surface, a process readily observed in the wild-type form (Burgueño et al., 2003). Our recent study employing a tandem three-step chromatography approach uncovered the impact of a single-residue substitution of a C-terminal cysteine, C394S, in reducing the receptor homo-oligomerization in vitro (Schonenbach et al., 2016). In the context of heteromerization, mass spectrometry and pull-down experiments have demonstrated that A_2A_R-D_2_R dimerization occurs via direct electrostatic interactions between the C-terminus of A_2A_R and the third intracellular loop of D_2_R (Ciruela et al., 2004). These results all suggest that the C-terminus may participate in A_2A_R oligomer formation. However, no studies to date have directly and systematically investigated the role of the C-terminus, or any IDRs, in GPCR oligomerization.

This study focuses on the homo-oligomerization of the human adenosine A_2A_R, a model GPCR, and seeks to address (i) whether the C-terminus engages in A_2A_R oligomerization, and if so, (ii) whether the C-terminus forms multiple oligomeric interfaces. We use size-exclusion chromatography (SEC) to assess the oligomerization levels of A_2A_R variants with strategic C-terminal modifications: mutations of a cysteine residue C394 and a cluster of charged residues ^355^ERR^357^, as well as systematic truncations at eight different sites along its length. We complemented our experimental study with an independent molecular dynamics (MD) simulation study of A_2A_R dimers of five C-terminally truncated A_2A_R variants designed to mirror the experimental constructs. We furthermore examined the oligomerization level of select C-terminally modified A_2A_R variants under conditions of varying ionic strength ranging from 0.15 to 0.95 M. To verify whether the A_2A_R oligomer populations are thermodynamic products, we performed a series of SEC analyses on SEC-separated monomer and dimer/oligomer populations to observe their repopulation into monomer and dimer/oligomer populations. Finally, to test whether the C-termini directly and independently promote A_2A_R oligomerization, we recombinantly expressed the entire A_2A_R C-terminal segment sans the transmembrane portion of the receptor and investigated its solubility and assembly properties with increasing ion concentration and temperature. This is the first study designed to uncover the role of the intrinsically disordered C-terminus on the oligomerization of a GPCR.

## Results

This study systematically investigates the role of the C-terminus on A_2A_R oligomerization and the nature of the involved interactions through strategic mutations and truncations at the C-terminus as well as modulation of the ionic strength of solvent. All experiments were done at 4°C unless stated otherwise. The experimental assessment of A_2A_R oligomerization relies on SEC analysis.

### SEC quantifies A_2A_R oligomerization

**Figure 1.**
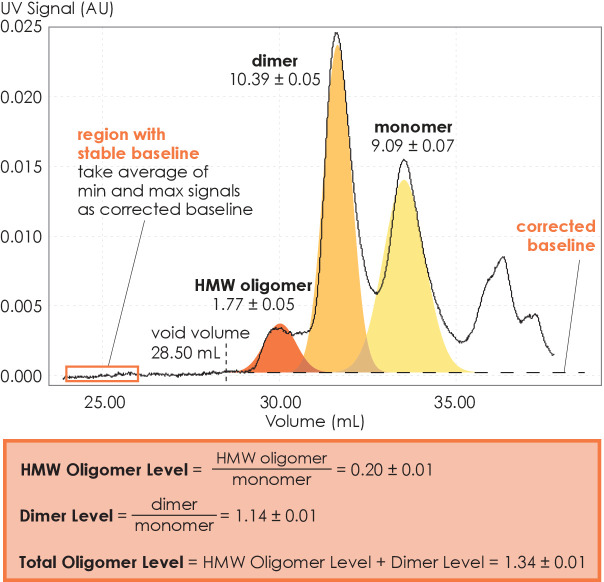
Method for collecting size-exclusion chromatography (SEC) data and assessing A_2A_R oligomerization. The SEC data is recorded every second as absorbance at 280 nm. The baseline is corrected to ensure uniform fitting and integration across the peaks. The areas under the curve, resulting from a multiple-Gaussian curve fit, express the population of each oligomeric species. The reported standard errors of integration are within a 95% confidence interval and are calculated from the variance of the fit, not experimental errors. The levels of high-molecular-weight oligomer and dimer are expressed relative to the monomeric population in arbitrary units. A representative calculation defining the oligomer levels is given in the box.

We performed SEC analysis on a mixture of ligand-active A_2A_R purified from a custom synthesized antagonist affinity column (Figure 1—figure supplement 1A). Distinct oligomeric species were separated and eluted in the following order: high-molecular-weight (HMW) oligomer, dimer, and monomer (Figure 1 and Figure 1—figure supplement 1B). This peak assignment has been verified with SEC-MALS (multi-angle light scattering) experiments, as detailed in a previous publication (Schonenbach et al., 2016). The population of each oligomeric species was quantified as the integral of each Gaussian from a multiple-Gaussian curve fit of the SEC signal. The reported standard errors were calculated from the variance of the fit that do not correspond to experimental errors (see Supplementary file 1 and Figure 1—figure supplement 2 for SEC data corresponding to all A_2A_R variants in this study). As this study sought to identify the factors that promote A_2A_R oligomerization, the populations with oligomeric interfaces (i.e., dimer and HMW oligomer) were compared with those without such interfaces (i.e., monomer). Hence, the populations of the HMW oligomer and dimer were expressed relative to the monomer population in arbitrary units as monomer-equivalent concentration ratios, henceforth referred to as population levels (Figure 1).

**Figure 1—figure supplement 1.**
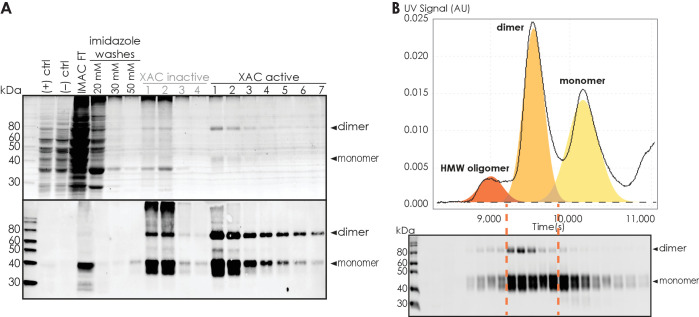
The purity and identity of A_2A_R are confirmed with total protein stain and western blot. (**A**) Representative total protein stain (upper panel) and western blot (lower panel) of A_2A_R-WT during purification. Positive ([+] ctrl) and negative ([–] ctrl) controls consist of 5 OD cell lysate of *Saccharomyces cerevisiae* BJ5464 cells expressing and not expressing A_2A_R WT, respectively. ‘IMAC FT’ indicates the flow-through from IMAC step. ‘XAC inactive’ and ‘XAC active’ indicate the fractions that do not and do bind to XAC during the ligand-affinity chromatography step. (**B**) Representative western blot of A_2A_R-WT during size-exclusion chromatography (SEC) separation. The fractions are matched to the distinct oligomeric peaks in the SEC chromatogram. Each lane on the blot is from 0.5 mL fractions eluted from a Superdex 200 10/300 GL (GE Healthcare) column. MagicMark protein ladder (LC5602) is used as the molecular weight standard. Figure 1—figure supplement 1—source data 1.Raw representative total protein stain of A_2A_R-WT during purification. Figure 1—figure supplement 1—source data 2.Labeled representative total protein stain of A_2A_R-WT during purification.Positive ([+] ctrl) and negative ([–] ctrl) controls consist of 5 OD cell lysate of *Saccharomyces cerevisiae* BJ5464 cells expressing and not expressing A_2A_R WT, respectively. ‘IMAC FT’ indicates the flow-through from IMAC step. ‘XAC inactive’ and ‘XAC active’ indicate the fractions that do not and do bind to XAC during the ligand-affinity chromatography step. MagicMark protein ladder (LC5602) is used as the molecular weight standard. Figure 1—figure supplement 1—source data 3.Raw representative western blot of A_2A_R-WT during purification. Figure 1—figure supplement 1—source data 4.Labeled representative western blot of A_2A_R-WT during purification.Positive ([+] ctrl) and negative ([–] ctrl) controls consist of 5 OD cell lysate of *Saccharomyces cerevisiae* BJ5464 cells expressing and not expressing A_2A_R WT, respectively. ‘IMAC FT’ indicates the flow-through from IMAC step. ‘XAC inactive’ and ‘XAC active’ indicate the fractions that do not and do bind to XAC during the ligand-affinity chromatography step. MagicMark protein ladder (LC5602) is used as the molecular weight standard. Figure 1—figure supplement 1—source data 5.Raw representative western blot of A_2A_R-WT during size-exclusion chromatography separation. Figure 1—figure supplement 1—source data 6.Labeled representative western blot of A_2A_R-WT during size-exclusion chromatography separation.Each lane on the blot is from 0.5 mL fractions eluted from a Superdex 200 10/300 GL (GE Healthcare) column. MagicMark protein ladder (LC5602) is used as the molecular weight standard.

**Figure 1—figure supplement 2.**
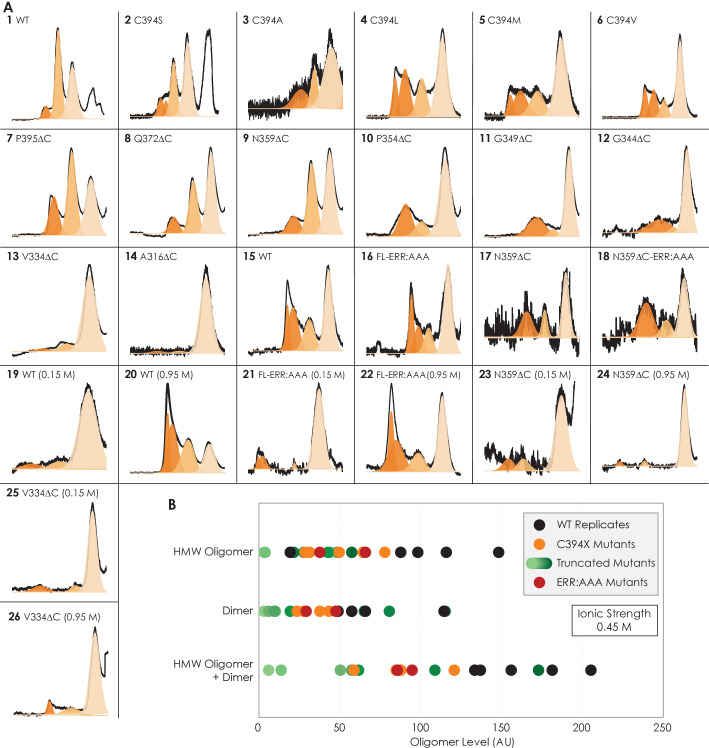
Size-exclusion chromatographic traces and data distribution of all A2AR variants used in the main text of this study. (**A**) Curve fitting using OriginLab of all A_2A_R variants used in the main text of this study, listed by the order they appear. By default, each oligomeric peak is fitted with one curve using Gaussian distribution and displayed by different color shades, with the high-molecular-weight (HMW) oligomer eluted first (dark orange), followed by the dimer (lighter orange), followed by the monomer (lightest orange). However, the HMW oligomer peak in some cases cannot be fitted with one curve and thus is fitted with two curves instead. This discrepancy can be explained by variation in HMW oligomerization order among the variants. The identity of each peak is confirmed with western blotting. The value and error from the curve fitting of each peak are given in Supplementary file 1. (**B**) Data distribution of all variants used in this study in comparison to five experimental replicates of A_2A_R-WT. The C-terminally truncated mutants are represented by different shades of green in increasing darkness corresponding to the increased length of the C-terminus, with the lightest shade representing the mutant with the shortest C-terminus (A316ΔC) and the darkest shade for the mutant with the longest C-terminus (P395ΔC). The levels of dimer and HMW oligomer are expressed relative to the monomeric population in arbitrary unit, with reported errors calculated from the variance of the fit, not experimental variation. There are significant variations in the dimer and HMW oligomer levels among the WT replicates, stemming from experimental errors. These variations are mitigated when the two parameters are added as the data distribution becomes more uniform. Also, the oligomerization levels of the WT replicates are consistently higher than the mutated and truncated variants. Figure 1—figure supplement 2—source data 1.Raw size-exclusion chromatography data of five experimental replicates of A_2A_R-WT.

### C-terminal amino acid residue C394 contributes to A_2A_R oligomerization

**Figure 2.**
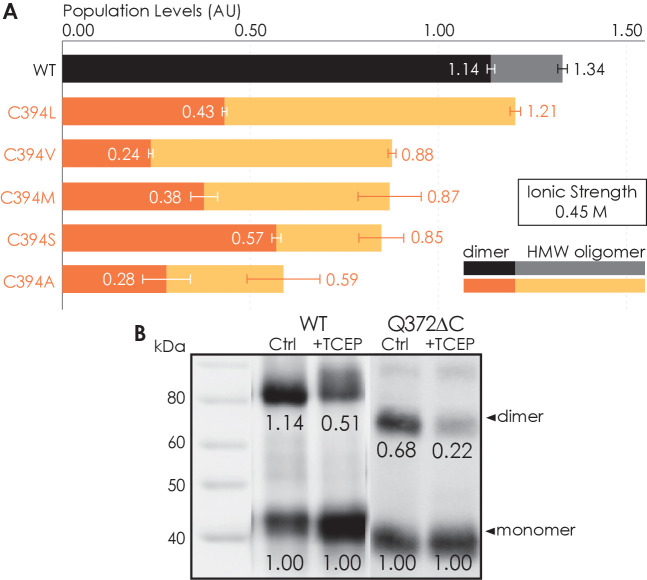
Residue C394 helps stabilize A_2A_R oligomerization via disulfide bonds. (**A**) The effect of C394X substitutions on A_2A_R oligomerization. The levels of dimer (dark colors) and high-molecular-weight oligomer (light colors) are expressed relative to the monomeric population in arbitrary units, with reported errors calculated from the variance of the fit, not experimental variation. (**B**) Line densitometry of western blot bands on size-exclusion chromatography (SEC)-separated dimeric populations of A_2A_R-WT and Q372ΔC with and without 5 mM TCEP. The level of dimer is expressed relative to the monomeric population in arbitrary units similarly to the SEC analysis. MagicMark protein ladder (LC5602) is used as the molecular weight standard. Figure 2—source data 1.Raw western blot of size-exclusion chromatography-separated dimeric populations of A_2A_R-WT with and without 5 mM TCEP. Figure 2—source data 2.Raw western blot of size-exclusion chromatography-separated dimeric populations of A_2A_R-WT with and without 5 mM TCEP.MagicMark protein ladder (LC5602) is used as the molecular weight standard. Figure 2—source data 3.Raw western blot of size-exclusion chromatography-separated dimeric populations of A_2A_R-Q372ΔC with and without 5 mM TCEP. Figure 2—source data 4.Raw western blot of size-exclusion chromatography-separated dimeric populations of A_2A_R-Q372ΔC with and without 5 mM TCEP.MagicMark protein ladder (LC5602) is used as the molecular weight standard. Figure 2—source data 5.Raw size-exclusion chromatography data of A_2A_R-WT and C394X variants.

To investigate whether the C-terminus of A_2A_R is involved in receptor oligomerization, we first examined the role of residue C394 as a previous study demonstrated that the mutation C394S dramatically reduced A_2A_R oligomer levels (Schonenbach et al., 2016). The C394S mutation was replicated in our experiments, alongside other amino acid substitutions for the cysteine, namely alanine, leucine, methionine, or valine, generating five A_2A_R-C394X variants. The HMW oligomer and dimer levels of A_2A_R wild-type (WT) were compared with those of the A_2A_R-C394X variants. We found that the dimer level of A_2A_R-WT was significantly higher than that of the A_2A_R-C394X variants (WT: 1.14; C394X: 0.24–0.57; Figure 2A). A similar result, though less pronounced, was observed when the HMW oligomer and dimer levels were considered together (WT: 1.34; C394X: 0.59–1.21; Figure 2A). This suggests that residue C394 plays a role in A_2A_R oligomerization, and even more prominently in A_2A_R dimerization.

To test whether residue C394 stabilizes A_2A_R dimerization by forming disulfide linkages, we incubated the SEC-separated dimers of A_2A_R-WT and A_2A_R-Q372ΔC with 5 mM of the reducing agent TCEP, followed by SDS-PAGE and western blotting. The population of each species was determined as the area under the densitometric trace. The dimer level was then expressed as monomer-equivalent concentration ratios in a manner similar to that of the SEC experiment described above. Upon incubation with TCEP, the dimer level of the A_2A_R-WT sample decreased from 1.14 to 0.51 (Figure 2B). This indicates that disulfide bond formation via residue C394 is one possible mechanism for A_2A_R dimerization. Interestingly, the dimer level of the A_2A_R-Q372ΔC sample also decreased from 0.68 to 0.22 (Figure 2B). This suggests that there may exist other inter-A_2A_R disulfide bonds that do not involve residue C394. Still, in both cases, a clearly visible population of A_2A_R dimer persists, even after reduction of disulfide bonds via TCEP (Figure 2B), suggesting that there must be additional interfacial sites that help drive A_2A_R dimer/oligomerization.

### C-terminus truncation systematically reduces A_2A_R oligomerization

**Figure 3.**
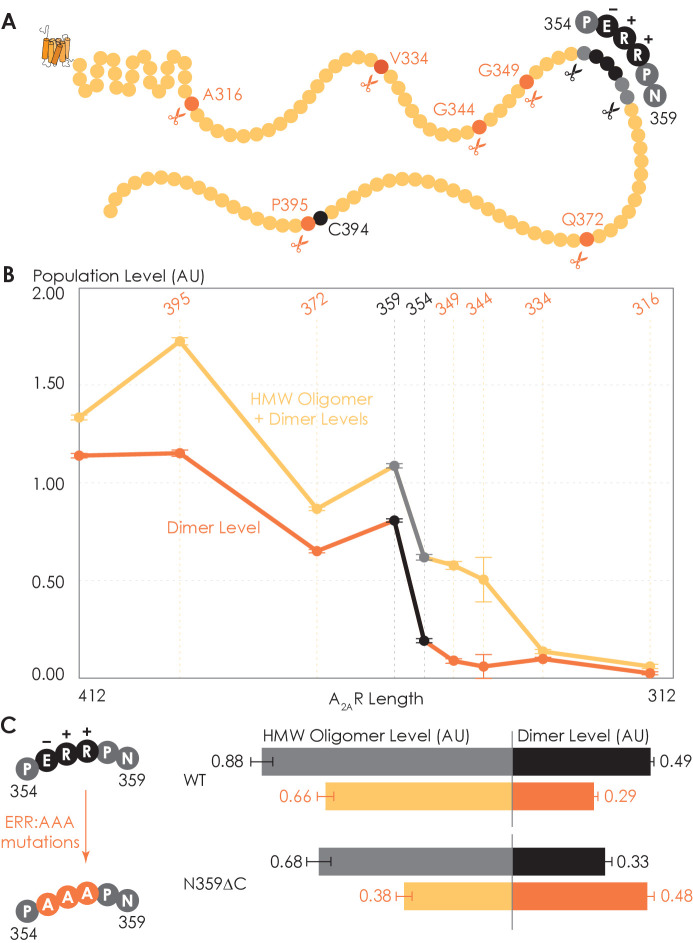
Truncating the C-terminus systematically affects A_2A_R oligomerization. (**A**) Depiction of where the truncation points are located on the C-terminus, with region 354–359 highlighted (in black) showing critical residues. (**B**) The levels of dimer and high-molecular-weight (HMW) oligomer are expressed relative to the monomeric population as an arbitrary unit and plotted against the residue number of the truncation sites, with reported errors calculated from the variance of the fit, not experimental variation. Region 354–359 is emphasized (in black and gray) due to a drastic change in the dimer and HMW oligomer levels. (**C**) The dependence of A_2A_R oligomerization on three consecutive charged residues ^355^ERR^357^. The substitution of residues ^355^ERR^357^ to ^355^AAA^357^ is referred to as the ERR:AAA mutations. The levels of dimer and HMW oligomer are expressed relative to the monomeric population as an arbitrary unit, with reported errors calculated from the variance of the fit, not experimental variation. Figure 3—source data 1.Raw size-exclusion chromatography data of A_2A_R-WT and C-terminally truncated ΔC variants.

To determine which interfacial sites in the C-terminus other than the disulfide-bonded cysteines drive A_2A_R dimer/oligomerization, we carried out systematic truncations at eight sites along the C-terminus (A316, V334, G344, G349, P354, N359, Q372, and P395), generating eight A_2A_R-ΔC variants (Figure 3A). The A_2A_R-A316ΔC variant corresponds to the removal of the entire disordered C-terminal region and is used in all published structural studies of A_2A_R (Martynowycz et al., 2020; Song et al., 2020; García-Nafría et al., 2018; Sun et al., 2017; Carpenter et al., 2016; Hino et al., 2012; Xu et al., 2011; Lebon et al., 2011; Doré et al., 2011; Jaakola et al., 2008; Fanelli and Felline, 2011). Using the SEC analysis described earlier (Figure 1), we evaluated the HMW oligomer and dimer levels of the A_2A_R-ΔC variants relative to that of the A_2A_R full-length-wild-type (FL-WT) control. Both the dimer and the total oligomer levels of A_2A_R decreased progressively with the shortening of the C-terminus, with almost no oligomerization detected upon complete truncation of the C-terminus at site A316 (Figure 3B). This result shows that the C-terminus drives A_2A_R oligomerization, with multiple potential interaction sites positioned along much of its length.

Interestingly, there occurred a dramatic decrease in the dimer level between the N359 and P354 truncation sites, from a value of 0.81 to 0.19, respectively (Figure 3B). A similar result, though less pronounced, was observed on the total oligomer level, with a decrease from 1.09 to 0.62 for the N359 and P354 truncation sites, respectively (Figure 3B). Clearly, the C-terminal segment encompassing residues 354–359 (highlighted in black in Figure 3A) is a key constituent of the A_2A_R oligomeric interface.

Since segment 354–359 contains three consecutive charged residues (^355^ERR^357^; Figure 3A), which could be involved in electrostatic interactions, we hypothesized that this ^355^ERR^357^ cluster could strengthen inter-protomer A_2A_R-A_2A_R association. To test this hypothesis, residues ^355^ERR^357^ were substituted by ^355^AAA^357^ on A_2A_R-FL-WT and A_2A_R-N359ΔC to generate A_2A_R-ERR:AAA variants (Figure 3C). We then compared the HMW oligomer and dimer levels of the resulting variants with controls (same A_2A_R variants but without the ERR:AAA mutations). We found that the ERR:AAA mutations had varied effects on the dimer level: decreasing for A_2A_R-FL-WT (ctrl: 0.49; ERR:AAA: 0.29) but increasing for A_2A_R-N359ΔC (ctrl: 0.33; ERR:AAA: 0.48) (Figure 3C). In contrast, the ERR:AAA mutations reduced the HMW oligomer level of both A_2A_R-FL-WT (ctrl: 0.88; ERR:AAA: 0.66) and A_2A_R-N359ΔC (ctrl: 0.68; ERR:AAA: 0.38) (Figure 3C). Consistently, the ERR:AAA mutation lowered the total oligomer level of both A_2A_R-FL-WT (ctrl: 1.37; ERR:AAA: 0.94) and A_2A_R-N359ΔC (ctrl: 1.01; ERR:AAA: 0.85) (Figure 3C). These results suggest that the charged residues ^355^ERR^357^ participate in A_2A_R oligomerization, with a greater effect in the context of a longer C-terminus and for forming higher-order oligomers. The question then arises as to what types of interactions are formed along the C-terminus that help stabilize A_2A_R oligomerization.

### C-terminus truncation disrupts complex network of non-bonded interactions necessary for A_2A_R dimerization

**Figure 4.**
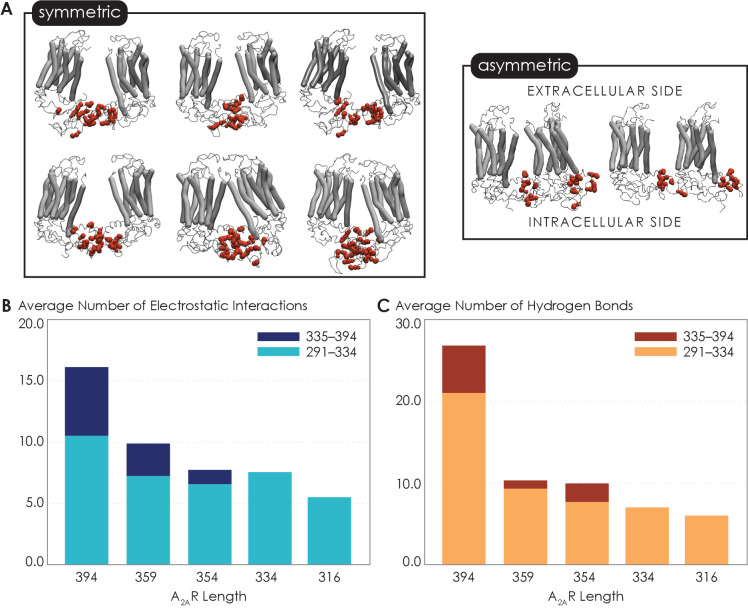
Non-bonded interactions of the extended C-terminus of A_2A_R play a critical role in stabilization of the dimeric interface. (**A**) Dimer configurations from cluster analysis in GROMACS of the 394-residue variant identify two major clusters involving either (1) the C-terminus of one protomer and the C-terminus, ICL2, and ICL3 of the second protomer or (2) the C-terminus of one protomer and ICL2, ICL3, and ECL2 of the second protomer. Spheres: residues forming intermolecular electrostatic contacts. (**B**) Average number of residues that form electrostatic contacts as a function of sequence length of A_2A_R. (**C**) Average number of residues that form hydrogen bonds as a function of sequence length of A_2A_R. The criteria for designating inter-A_2A_R contacts as electrostatic interactions or hydrogen bonds are described in detail in Materials and methods. Figure 4—source data 1.Detailed data regarding the multiple interfaces of A_2A_R and the network of non-bonded interactions that stabilize these interfaces.(**A**) Dimer configurations from cluster analysis in GROMACS of the 394-residue variant. (**B**) Average number of residues that form electrostatic contacts as a function of sequence length of A_2A_R. (**C**) Average number of residues that form hydrogen bonds as a function of sequence length of A_2A_R.

Given that the structure of A_2A_R dimers or oligomers is unknown, we next used MD simulations to seek molecular-level insights into the role of the C-terminus in driving A_2A_R dimerization and to gain an understanding of what types of interactions and sites may be involved in this process. First, to explore A_2A_R dimeric interface, we performed coarse-grained (CG) MD simulations using the Martini force field (see Materials and methods for details). The Martini force field can access the length and time scales relevant to membrane protein oligomerization, albeit at the expense of atomic-level details. We carried out a series of CGMD simulations on five A_2A_R-ΔC variants designed to mirror the experiments by systematic truncation at five sites along the C-terminus (A316, V334, P354, N359, and C394). Our results revealed that A_2A_R dimers were formed with multiple interfaces, all involving the C-terminus only (Figure 4A). The transmembrane heptahelical bundles were not a part of the dimeric interfaces as they all showed distances greater than the minimum distance criterion of 7 Å for interacting helices. The vast majority of A_2A_R dimers were symmetric, with the C-termini of the protomers directly interacting with each other. A smaller fraction of the dimers had asymmetric orientations, with the C-terminus of one protomer interacting with other parts of the other protomer, such as ICL2 (the second intracellular loop) and ICL3 (Figure 4A).

Our observation of multiple A_2A_R oligomeric interfaces, which is consistent with previous studies (Fanelli and Felline, 2011; Song et al., 2020), suggests that tunable, non-covalent intermolecular interactions may be involved in receptor dimerization. We first dissected two key non-covalent interaction types: electrostatic and hydrogen bonding interactions. Electrostatic interactions were calculated from CGMD simulations, while hydrogen bonds were quantified from atomistic MD simulation as the CG model merges all hydrogens into a CG bead and hence cannot report on hydrogen bonds. This analysis was performed on the symmetric dimers as they constituted the more dominant population. With the least truncated A_2A_R variant containing the longest C-terminus, A_2A_R-C394ΔC, we observed an average of 15.9 electrostatic contacts (Figure 4B) and 26.7 hydrogen bonds (Figure 4C) between the C-termini of the protomers. This result shows that both electrostatic interactions and hydrogen bonds can play important roles in A_2A_R dimer formation.

Upon further C-terminus truncation, the average number of both electrostatic contacts and hydrogen bonds involving C-terminal residues progressively declined, respectively reaching 5.4 and 6.0 for A_2A_R-A316ΔC (in which the disordered region of the C-terminus is removed) (Figure 4B, C). This result is consistent with the experimental result, which demonstrated a progressive decrease of A_2A_R oligomerization with the shortening of the C-terminus (Figure 3B). Interestingly, upon systematic truncation of the C-terminal segment 335–394, we observed in segment 291–334 a steady decrease in the average number of electrostatic contacts, from 10.4 to 7.4 (Figure 4B). This trend was even more pronounced with hydrogen bonding contacts involving segment 291–334 decreasing drastically from 21.0 to 7.0 as segment 335–394 was gradually removed (Figure 4C). This observation that truncation of a C-terminal segment reduces inter-A_2A_R contacts elsewhere along the C-terminus indicates that an allosteric mechanism of dimerization exists, in which an extended C-terminus of A_2A_R stabilizes inter-A_2A_R interactions near the heptahelical bundles of the dimeric complex. These results demonstrate that A_2A_R dimers can be formed via multiple interfaces and stabilized by an allosteric network of electrostatic interactions and hydrogen bonds along much of its C-terminus.

### Ionic strength modulates oligomerization of C-terminally truncated A_2A_R variants

So far, we have demonstrated that the C-terminus clearly plays a role in forming A_2A_R oligomeric interfaces. However, it remains unclear what the driving factors of A_2A_R oligomerization are and whether the oligomeric populations are thermodynamic products. The variable nature of A_2A_R oligomeric interfaces suggests that the main driving forces must be non-covalent interactions, such as electrostatic interactions and hydrogen bonds. Modulating the solvent ionic strength is an effective method to identify the types of non-covalent interaction(s) at play. Specifically, with increasing ionic strength, electrostatic interactions are weakened (based on Debye–Hückel theory, most electrostatic bonds at a distance greater than 5 Å are screened out at an ionic strength of 0.34 M at 4°C) and depletion interactions are enhanced with salting-out salts, while hydrogen bonds remain relatively impervious. For this reason, we subjected various A_2A_R variants (FL-WT, FL-ERR:AAA, N359ΔC, and V334ΔC) to ionic strength ranging from 0.15 to 0.95 M by adding NaCl (buffer composition shown in Materials and methods). The HMW oligomer and dimer levels of the four A_2A_R variants were determined and plotted as a function of ionic strengths.

**Figure 5.**
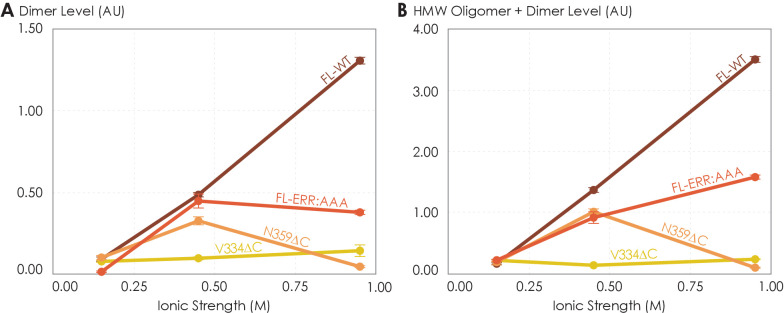
The effects of ionic strength on the oligomerization of various A_2A_R variants reveal the involvement of depletion interactions. The levels of (**A**) dimer and (**B**) high-molecular-weight oligomer + dimer are expressed relative to the monomeric population as an arbitrary unit and plotted against ionic strength, with reported errors calculated from the variance of the fit, not experimental variation. NaCl concentration is varied to achieve ionic strengths of 0.15, 0.45, and 0.95 M. Figure 5—source data 1.Raw size-exclusion chromatography data of various A_2A_R variants under different ionic strengths of 0.15, 0.45, and 0.95 M.

The low ionic strength of 0.15 M should not affect hydrogen bonds or electrostatic interactions if present. We found that the dimer and total oligomer levels of all four variants were near zero (Figure 5). This is a striking experimental observation: despite being shown to play a role in stabilizing A_2A_R dimers according to our MD simulations (Figure 4B, C), we can conclude that electrostatic and hydrogen-bonding interactions are not the dominant driving force for A_2A_R association. The question remains whether depletion interactions could facilitate A_2A_R oligomerization.

**Figure 5—figure supplement 1.**
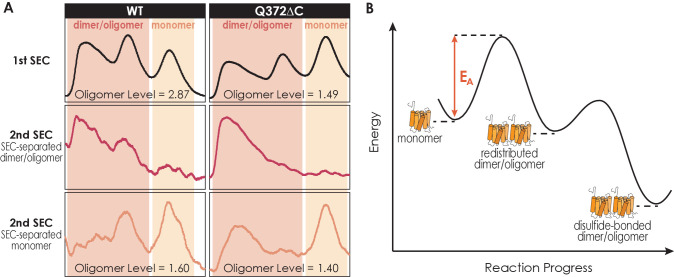
The dimer/oligomerization of A_2A_R is a thermodynamic process where the dimer and high-molecular-weight oligomer once formed are kinetically trapped. (**A**) Size-exclusion chromatography (SEC) chromatograms of the consecutive rounds of SEC performed on A_2A_R-WT and Q372ΔC. The first rounds of SEC are to separate the dimer/oligomer population and the monomer population, while the second rounds of SEC are performed on these SEC-separated populations to assess their stability and reversibility. The total oligomer level is expressed relative to the monomeric population in arbitrary units. (**B**) Energy diagram depicting A_2A_R oligomerization progress. The monomer needs to overcome an activation barrier (E_A_), driven by depletion interactions, to form the dimer/oligomer. Once formed, the dimer/oligomer populations are kinetically trapped by disulfide linkages. Figure 5—figure supplement 1—source data 1.Raw size-exclusion chromatography (SEC) data of the consecutive rounds of SEC performed on A_2A_R-WT and Q372ΔC.

At higher ionic strengths of 0.45 M and 0.95 M, the dimer and total oligomer levels of A_2A_R-V334ΔC still remained near zero (Figure 5). In contrast, we observed a progressive and significant increase in the dimer and total oligomer levels of A_2A_R-FL-WT with increasing ionic strength (Figure 5). This result indicates that A_2A_R oligomerization is driven by depletion interactions enhanced with increasing ionic strength and that these interactions must involve the C-terminal segment after residue V334.

Upon closer examination, we recognize that at the very high ionic strength of 0.95 M the increase in the dimer and total oligomer levels was robust for A_2A_R-FL-WT, but less pronounced for A_2A_R-FL-ERR:AAA (Figure 5). Furthermore, this high ionic strength even had an opposite effect on A_2A_R-N359ΔC, with both its dimer and total oligomer levels abolished (Figure 5). These results indicate that the charged cluster ^355^ERR^357^ and the C-terminal segment after residue N359 promote the depletion interactions to drive A_2A_R oligomerization. Taken together, we can conclude that A_2A_R oligomerization is more robust when the C-terminus is fully present and the ionic strength higher, suggesting that depletion interactions via the C-terminus are strong driving factors of A_2A_R oligomerization.

The discussion of depletion interactions as driving factors assumes that A_2A_R dimer/oligomer populations are thermodynamic products at equilibrium with the A_2A_R monomer population. However, some of the A_2A_R dimer/oligomer populations may be kinetically stabilized. To address this question, we tested the stability and reversibility of A_2A_R oligomers by performing a second round of SEC on the monomer and dimer/oligomer populations of the A_2A_R-WT and Q372ΔC variants. We found that the SEC-separated monomers repopulate into dimer/oligomer, with the total oligomer level after redistribution comparable with that of the initial samples for both A_2A_R-WT (initial: 2.87; redistributed: 1.60) and Q372ΔC (initial: 1.49; redistributed: 1.40) (Figure 5—figure supplement 1A). This observation indicates that A_2A_R oligomer is a thermodynamic product with a lower free energy compared with that of the monomer (Figure 5—figure supplement 1B). This agrees with the results we have shown in Supplementary file 1 that the oligomer levels of A_2A_R-WT are consistent among replicates (1.34–2.05) and that A_2A_R oligomerization can be modulated with ionic strengths via depletion interactions (Figure 5).

In contrast, the SEC-separated dimer/oligomer populations do not repopulate to form monomers (Figure 5—figure supplement 1A). This observation is consistent with a published study of ours on A_2A_R dimers (Schonenbach et al., 2016), indicating that once the oligomers are formed, some are kinetically trapped and thus cannot redistribute into monomers. We believe that disulfide linkages are likely candidates to kinetically stabilize A_2A_R oligomers, as demonstrated by their redistribution into monomers only in the presence of a reducing agent (Figure 2B).

Taken together, we suggest that A_2A_R oligomerization is a thermodynamic process (Figure 5—figure supplement 1B), with the free energy of the dimer/oligomers lowered by depletion forces that hence increase their population relative to that of the monomers (there always exists a distribution between the two). Once formed, the redistributed dimer/oligomer populations may be kinetically stabilized by disulfide linkages. The question then arises whether inter-A_2A_R interactions are primarily a result of the C-termini directly interacting with one another. This question motivated us to carry out a study focused on investigating the behavior of A_2A_R C-terminus sans the transmembrane domains.

### The isolated A_2A_R C-terminus is prone to aggregation

To test whether A_2A_R oligomerization is driven by direct depletion interactions among the C-termini of the protomers, we assayed the solubility and assembly properties of the stand-alone A_2A_R C-terminus—an intrinsically disordered peptide—sans the upstream transmembrane regions. Since depletion interactions can be manifested via the hydrophobic effect (van der Vegt et al., 2017), we examined whether this effect can also drive the assembly of the A_2A_R C-terminal peptides.

**Figure 6.**
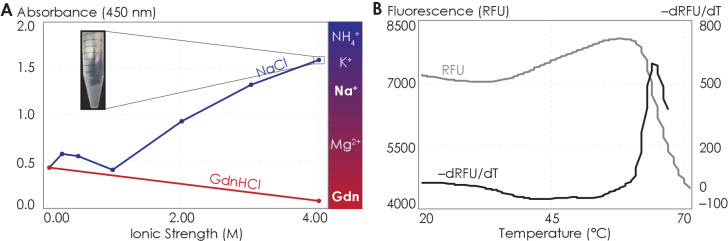
The A_2A_R C-terminus is prone to aggregation. (**A**) Absorbance at 450 nm of the A_2A_R C-terminus in solution, with NaCl and GdnHCl concentrations varied to achieve ionic strengths 0–4 M. Inset: the solution at ionic strength 4 M achieved with NaCl. The Hofmeister series is provided to show the ability of cations to salt-out (blue) or salt-in (red) proteins. (**B**) SYPRO orange fluorescence of solutions containing the A_2A_R C-terminus as the temperature was varied from 20°C to 70°C (gray). The change in fluorescence, measured in relative fluorescence unit (RFU), was calculated by taking the first derivative of the fluorescence curve (black). Figure 6—source data 1.Detailed data showing the propensity of A_2A_R C-terminus to aggregate.(**A**) Absorbance at 450 nm of the A_2A_R C-terminus in solution, with NaCl and GdnHCl concentrations varied to achieve ionic strengths 0–4 M. (**B**) SYPRO orange fluorescence of solutions containing the A_2A_R C-terminus as the temperature was varied from 20°C to 70°C (gray). The change in fluorescence, measured in relative fluorescence unit (RFU), was calculated by taking the first derivative of the fluorescence values.

It is an active debate whether the hydrophobic effect can be promoted or suppressed by ions with salting-out or salting-in tendency, respectively (Thomas and Elcock, 2007; Graziano, 2010; Zangi et al., 2007; Grover and Ryall, 2005). We increased the solvent ionic strength using either sodium (salting-out) or guanidinium (salting-in) ions and assessed the aggregation propensity of the C-terminal peptides using UV-Vis absorption at 450 nm, which indicates the turbidity of the solution. We first observed the behavior of the C-terminus with increasing salting-out NaCl concentrations. At NaCl concentrations below 1 M, the peptide was dominantly soluble, despite showing slight aggregation at NaCl concentrations between 250 and 500 mM (Figure 6A). At NaCl concentrations above 1 M, A_2A_R C-terminal peptides strongly associated into insoluble aggregates (Figure 6A). Consistent with the observations made with the intact receptor (Figure 5), the A_2A_R C-terminus showed the tendency to progressively associate and eventually precipitate with increasing ionic strengths, suggesting that depletion interactions drive the association and precipitation of the peptides. We next observed the behavior of the C-terminus with increasing concentrations of guanidine hydrochloride (GdnHCl), which contains salting-in cations that do not induce precipitation and instead facilitate the solubilization of proteins (Heyda et al., 2017; Baldwin, 1996). Our results demonstrated that the A_2A_R C-terminus incubated in 4 M GdnHCl showed no aggregation propensity (Figure 6A), validating our expectation that salting-in salts do not enhance depletion interactions. These observations demonstrate that the C-terminal peptide in and of itself, outside the context of the lipid membrane and TM domain, can directly interact with other C-terminal peptides to form self-aggregates in the presence of ions, and presumably solutes, that have salting-out effects.

**Figure 6—figure supplement 1.**
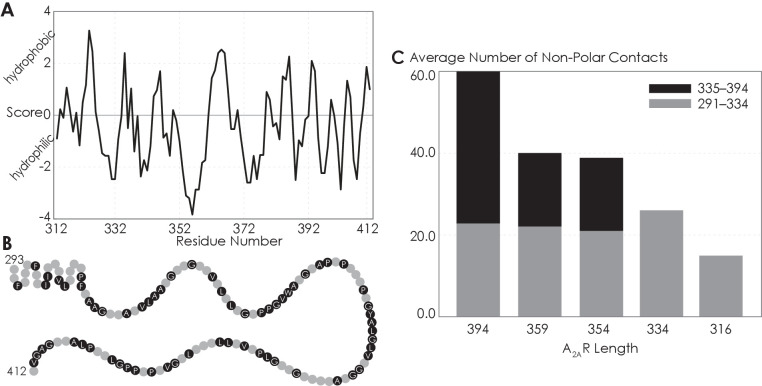
The C-terminus of A_2A_R can form non-polar contacts. (**A**) Hydropathy plot against A_2A_R residue number showing the hydrophobicity of A_2A_R C-terminus, scored with ProtScale using method described by Kyte and Doolittle, window size of 3. Positive scores represent hydrophobicity and negative scores hydrophilicity. (**B**) The non-polar residues in A_2A_R C-terminus. (**C**) Average number of residues that form non-polar contacts as a function of sequence length of A_2A_R. The criteria for designating inter-A_2A_R contacts as non-polar interactions are described in detail in Materials and methods. Figure 6—figure supplement 1—source data 1.Detailed data showing the ability of A_2A_R C-terminus to form non-polar contacts.(**A**) Hydropathy plot against A_2A_R residue number showing the hydrophobicity of A_2A_R. C-terminus, scored with ProtScale using method described by Kyte and Doolittle, window size of 3. Positive scores represent hydrophobicity and negative scores hydrophilicity. (**C**) Average number of residues that form non-polar contacts as a function of sequence length of A_2A_R.

Attractive hydrophobic interactions among the hydrophobic residues are further enhanced when the water that solvate the protein surface have more favorable interactions with other water molecules, ions, or solutes than with the protein surface, here the truncated C-terminus (Larsen et al., 1998; Tsai and Nussinov, 1997; Tsai et al., 1997). We explored the possible contribution of hydrophobic interactions to the aggregation of the C-terminal peptides using both experimental and computational approaches. Using differential scanning fluorimetry (DSF), we gradually increased the temperature to melt the C-terminal peptides, exposing any previously buried hydrophobic residues (Figure 6—figure supplement 1A, B), which then bound to the SYPRO orange fluorophore, resulting in an increase in fluorescence signal. Our results showed that as the temperature increased, a steady rise in fluorescence was observed (Figure 6B), indicating that multiple hydrophobic residues were gradually exposed to the SYPRO dye. However, at approximately 65°C, the melt peak signal was abruptly quenched (Figure 6B), indicating that the hydrophobic residues were no longer exposed to the dye. This observation suggests that, at 65°C, enough hydrophobic residues in the C-terminal peptides become exposed such that they collapse on one another (thus expelling the bound dye molecules), resulting in aggregation. This experimental result is further supported by our CGMD computational analysis of C-terminal non-polar contacts found in A_2A_R symmetrical dimers (Figure 6—figure supplement 1C). Specifically, we observed an average of 60 non-polar contacts for A_2A_R-C394ΔC. This number progressively declined upon further C-terminus truncation, reaching 15 for A_2A_R-A316ΔC. Clearly, the hydrophobic effect can cause A_2A_R C-terminal peptides to directly associate. These results demonstrate that A_2A_R oligomer formation can be driven by depletion interactions among the C-termini of the protomers by non-polar contacts.

## Discussion

The key finding of this study is that the C-terminus of A_2A_R, removed in all previously published structural studies, is directly responsible for receptor oligomerization. Using a combination of experimental and computational approaches, we demonstrate that the C-terminus stabilizes A_2A_R oligomers via a combination of disulfide linkages, hydrogen bonds, electrostatic interactions, and hydrophobic interactions. This diverse combination of interactions is greatly enhanced by depletion interactions, forming a network of malleable bonds that drives A_2A_R oligomerization and gives rise to multiple oligomeric interfaces.

Intermolecular disulfide linkages play a role in A_2A_R oligomerization, potentially by kinetically trapping the receptor oligomers. Among the seven cysteines that do not form intramolecular disulfide bonds (De Filippo et al., 2016; Naranjo et al., 2015; O'Malley et al., 2010), residue C394 is largely involved in stabilizing A_2A_R oligomers (Figure 2A). Indeed, this cysteine is highly conserved and a C-terminal cysteine is almost always present in A_2A_R homologs (Pándy-Szekeres et al., 2018), suggesting that it may serve an important role in vivo. There may also exist inter-A_2A_R disulfide linkages that do not involve residue C394 at all as the SEC-separated dimer/oligomer populations of A_2A_R-Q372ΔC, which lack residue C394, were still resistant to TCEP reduction (Figure 2B) and appear to be kinetically trapped (Figure 5—figure supplement 1). Such disulfide linkages may involve other cysteines in the hydrophobic core of A_2A_R, namely C28^1.54^, C82^3.30^, C128^4.49^, C185^5.46^, C245^6.47^, or C254^6.56^. Many examples exist where disulfide linkages help drive GPCR oligomerization, including the CaR-mGluR_1_ heterodimer (Gama et al., 2001), homodimers of mGluR_5_ (Romano et al., 1996), M_3_R (Zeng and Wess, 1999), V_2_R (Zhu and Wess, 1998), 5-HT_4_R (Berthouze et al., 2007) and 5**-**HT_1D_R (Lee et al., 2000), and even higher-order oligomers of D_2_R (Guo et al., 2008). Although unconventional cytoplasmic disulfide bonds have been reported (Saaranen and Ruddock, 2013; Locker and Griffiths, 1999), no study has shown how such linkages would be formed in vivo as the cytoplasm lacks the conditions and machinery required for disulfide bond formation (Gaut and Hendershot, 1993; Hwang et al., 1992; Helenius et al., 1992; Creighton et al., 1980).

**Figure 7.**
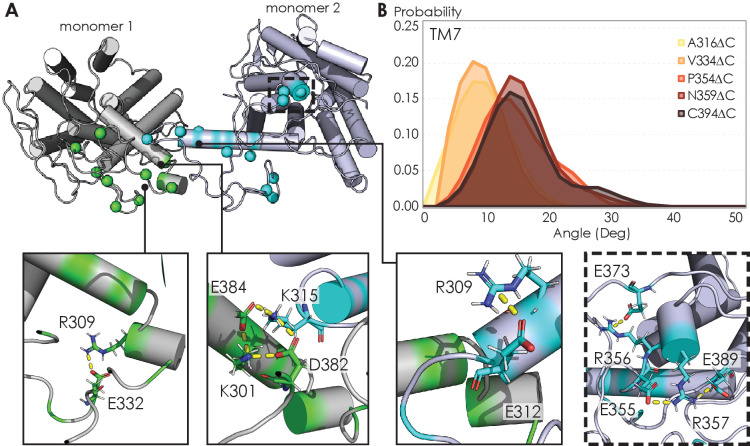
Visualizing A2AR dimeric interface and observing conformational changes of the TM7 using MD simulations. (**A**) Representative snapshot of A_2A_R-C394ΔC dimers shows salt bridge formation between a sample trajectory. The insets are closeups of the salt bridges, which can be both intra- and intermolecular. The last inset shows a network of salt bridges with the charged cluster ^355^ERR^357^ involved. (**B**) Helical tilt angles for TM7 helix in A_2A_R as a function of protein length. Systematic truncations of the C-terminus lead to rearrangement of the heptahelical bundle. The participation of the C-terminus in A_2A_R dimerization increases the tilting of the TM7 domain, which is in closest proximity to the C-terminus. Figure 7—source data 1.MD simulations data used to visualize A_2A_R dimeric interface and observe the conformational changes of the TM7.(**A**) List of all C-terminal residue pairs of A_2A_R-C394ΔC dimers engaging in electrostatic interactions. (**B**) Helical tilt angles for TM7 helix in A_2A_R as a function of protein length.

The electrostatic interactions that stabilize A_2A_R oligomer formation come from multiple sites along the C-terminus. From a representative snapshot of a A_2A_R-C394ΔC dimer from our MD simulations (Figure 7A), we could visualize not only the intermolecular interactions calculated from the CGMD simulations (Figure 4B), but also intramolecular salt bridges. In particular, the ^355^ERR^357^ cluster of charged residues lies distal from the dimeric interface but still forms several salt bridges (Figure 7A, inset). This observation is supported by our experimental results showing that substituting this charged cluster with alanines reduces the total A_2A_R oligomer levels (Figure 3C). However, it is unclear how such salt bridges involving this ^355^ERR^357^ cluster are enhanced by depletion interactions (Figure 5) as electrostatic interactions are usually screened out at high ionic strengths. In our MD simulations, we also observed networks of salt bridges along the dimeric interface, for example, between K315 of one monomer and D382 and E384 of the other monomer (Figure 7A, inset). The innate flexibility of the C-terminus could facilitate the formation of such salt bridges, which then help stabilize A_2A_R dimers.

**Figure 7—figure supplement 1.**
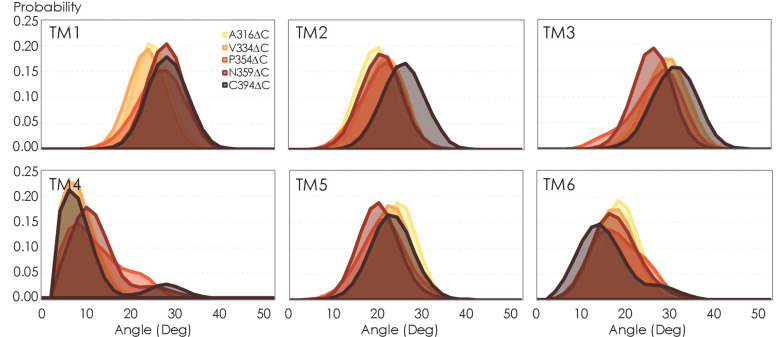
Helical tilt angles for TM1–6 helices in A_2A_R as a function of protein length. Systematic truncations of the C-terminus lead to rearrangement of the heptahelical bundle, propagated to the entire receptor and is especially pronounced in helices proximal to the C-terminus, that is, TM1, TM2, TM7. For almost all TM helices, a noticeable shift in tilt angle occurs upon modeling the full-length (394 residues) variant. This behavior is fundamentally different from the conventional model of G protein-coupled receptor (GPCR) activation, in which TM 1, 2, 4, and 7 remain rigid, with TM5 and TM6 undergoing an outward tilt/rotation to enable binding to the cognate G protein. Relaxation of the heptahelical bundle (i.e., an increase in helical tilt) as a function of protein length and dimerization could potentially be critical to our understanding of the activation mechanism of A_2A_R as past studies have overwhelmingly focused on activation of the monomer. Figure 7—figure supplement 1—source data 1.Helical tilt angles for TM1–6 helices in A_2A_R as a function of protein length.

Our finding that A_2A_R forms homo-oligomers via multiple interfaces (Figure 4A) agrees with the increasing number of studies reporting multiple and interconverting oligomeric interfaces in A_2A_R and other GPCRs (Song et al., 2020; Ghosh et al., 2014; Periole et al., 2012; Fanelli and Felline, 2011; Liu et al., 2012; Huang et al., 2013; Manglik et al., 2012; Thorsen et al., 2014; Fotiadis et al., 2006; Fotiadis et al., 2003; Liang et al., 2003; Xue et al., 2015; Dijkman et al., 2018). When translated to in vivo situations, GPCR oligomers can also transiently associate and dissociate (Kasai et al., 2018; Tabor et al., 2016; Möller et al., 2020; Vilardaga et al., 2008). Such conformational changes require that the oligomeric interfaces be formed by interactions that can easily be modulated. This is consistent with our study, which demonstrates that depletion interactions via the intrinsically disordered, malleable C-terminus drive A_2A_R oligomerization. Because depletion interactions can be readily tuned by environmental factors, such as ionic strength, molecular crowding, and temperature, the formation of GPCR oligomeric complexes could be dynamically modulated in response to environmental cues to regulate receptor function.

Not only did we find multiple A_2A_R oligomeric interfaces, we also found that these interfaces can be either symmetric or asymmetric. This finding is supported by a growing body of evidence that there exists both symmetric and asymmetric oligomeric interfaces for A_2A_R (Song et al., 2020) and many other GPCRs. Studies using various biochemical and biophysical techniques have shown that heterotetrameric GPCR complexes can be formed by dimers of dimers, including μOR-δOR (Golebiewska et al., 2011), CXC_4_R-CC_2_R (Armando et al., 2014), CB_1_R/D_2_R (Bagher et al., 2017), as well as those involving A_2A_R, such as A_1_R-A_2A_R (Navarro et al., 2018a; Navarro et al., 2016) and A_2A_R-D_2_R (Navarro et al., 2018b). The quaternary structures identified in these studies required specific orientations of each protomer, with the most viable model involving a stagger of homodimers with symmetric interfaces (DelaCuesta-Barrutia et al., 2020). On the other hand, since symmetric interfaces limit the degree of receptor association to dimers, the HMW oligomer of A_2A_R observed in this (Song et al., 2020) and other studies (Schonenbach et al., 2016; Vidi et al., 2008) can only be formed via asymmetric interfaces. It is indeed tempting to suggest that the formation of the HMW oligomer of A_2A_R may even arise from combinations of different interfaces. In any case, the wide variation of GPCR oligomerization requires the existence of both symmetric and asymmetric oligomeric interfaces.

The ultimate question to answer is how oligomerization alters A_2A_R function. In the case of A_2A_R, displacement of the transmembrane domains has been demonstrated to be the hallmark of receptor activation (Eddy et al., 2018; Sušac et al., 2018; Prosser et al., 2017; Ye et al., 2016), but no studies have linked receptor oligomerization with the arrangement of the TM bundles in A_2A_R. Our MD simulations revealed that C-terminus truncation resulted in structural changes in the heptahelical bundles of A_2A_R dimers. Specifically, as more of the C-terminus was preserved, we observed a progressive increase in the helical tilt of TM7 (Figure 7B). This change in helical tilt occurred for the entire heptahelical bundle, with an increase in tilt for TM1, TM2, TM3, TM5, and TM7, and a decrease in tilt for TM4 and TM6 (Figure 7—figure supplement 1). The longer C-terminus in the full-length A_2A_R permits greater rearrangements in the transmembrane regions, leading to the observed change in helical tilt. Furthermore, in the cellular context, it has been demonstrated that truncation of the C-terminus significantly reduced receptor association with Gα_s_ and cAMP production in cellular assays (Koretz et al., 2021). These results hint at potential conformational changes of A_2A_R upon oligomerization, necessitating future investigation on functional consequences.

Like all biophysical studies of membrane proteins in non-native environments, a drawback in our study is the question whether the above results, conducted in detergent micelles, can be translated to bilayer or cellular context. It has been demonstrated that the propensity of membrane proteins to associate and oligomerize is greater in lipid bilayers compared to that in detergent micelles (Popot and Engelman, 1990). Furthermore, in the cellular context, A_2A_R has been shown to assemble into homo-oligomers in transfected HEK293 cells (Canals et al., 2003) and in Cath.A differentiated neuronal cells (Vidi et al., 2008), while C-terminally truncated A_2A_R shows no protein aggregation or clustering on the cell surface, in contrast with its WT form (Burgueño et al., 2003). Therefore, we speculate that A_2A_R oligomerization will be present in the lipid bilayer and cellular environment. Regardless, given that most biophysical structure-function studies of GPCRs are conducted in detergent micelles and other artificial membrane mimetics, it is critical to understand the role of the C-terminus in the oligomerization of A_2A_R reconstituted in detergent micelles.

C-terminal truncations prior to crystallization and structural studies may be the main reason for the scarcity of GPCR structures featuring oligomers. In that context, this study offers valuable insights and approaches into how the oligomerization of A_2A_R and potentially of other GPCRs can be tuned by modifying the intrinsically disordered C-terminus and varying salt types and concentrations. The presence of A_2A_R oligomeric populations with partial C-terminal truncations means that one can now study its oligomerization with less perturbation from the C-terminus. We also present evidence that the multiple C-terminal interactions that drive A_2A_R oligomerization can be easily modulated by ionic strength and specific salts (Figures 5 and 6). Given that ~75% and ~15% of all class A GPCRs possess a C-terminus of >50 and >100 amino acid residues (Mirzadegan et al., 2003), respectively, it will be worthwhile to explore the prospect of tuning GPCR oligomerization not only by shortening the C-terminus but also with simpler approaches such as modulating ionic strength and the surrounding salt environment.

### Conclusion

This study emphasizes for the first time the definite impact of the C-terminus on A_2A_R oligomerization, which can be extended to include the oligomers formed by other GPCRs with a protracted C-terminus. We have shown that the oligomerization of A_2A_R is strongly driven by depletion interactions along the C-terminus, further modulating and enhancing the multiple interfaces formed via a combination of hydrogen, electrostatic, hydrophobic, and covalent disulfide interactions. The task remains to link A_2A_R oligomerization to functional roles of the receptor. From a structural biology standpoint, visualizing the multiple oligomeric interfaces of A_2A_R in the presence of the full-length C-terminus is key to investigating whether these interfaces give rise to different oligomer functions.

## Materials and methods

**Key resources table:** 

Reagent type (species) or resource	Designation	Source or reference	Identifiers	Additional information
Recombinant DNA reagent	pITy (plasmid)	Parekh et al., 1996		
Strain, strain background (*Saccharomyces cerevisiae*)	BJ5464	Robinson Lab – Carnegie Mellon University		
Strain, strain background (*Escherichia coli*)	BL21 (DE3)	Sigma, St. Louis, MO, USA	#CMC0014	
Chemical compound, drug	DDM	Anatrace, Maumee, OH, USA	#D310	
Chemical compound, drug	CHAPS	Anatrace, Maumee, OH, USA	#C216	
Chemical compound, drug	CHS	Anatrace, Maumee, OH, USA	#CH210	
Chemical compound, drug	Xanthine amine congener	Sigma, St. Louis, MO, USA	#X103	
Chemical compound, drug	Theophylline	Sigma, St. Louis, MO, USA	#T1633	
Commercial assay, kit	Affigel 10 resin	BioRad, Hercules, CA, USA	#1536099	
Commercial assay, kit	Tricorn Superdex 200 10/300 GL column	GE Healthcare, Pittsburgh, PA, USA	#17-5175-01	
Antibody	Anti-A_2A_R, clone 7F6-G5-A2 (Mouse monoclonal)	Millipore, Burlington, MA, USA	#05-717	(1:500) dilution
Antibody	Anti-Mouse IgG H&L DyLight 550 (Goat monoclonal)	Abcam, Cambridge, MA, USA	#ab96880	(1:600) dilution
Software, algorithm	MODELLER 9.23	Eswar et al., 2006		
Software, algorithm	martinize.py script	de Jong et al., 2013		
Software, algorithm	ELNeDyn elastic network	Periole et al., 2009		
Software, algorithm	MARTINI coarse-grained force field v2.2	Monticelli et al., 2008		
Software, algorithm	GROMACS 2016	Abraham et al., 2015		
Software, algorithm	backward.py script	Wassenaar et al., 2014		
Software, algorithm	LINCS	Hess et al., 1997		
Software, algorithm	CHARMM36 and TIP3P force fields	Best et al., 2012; Jorgensen et al., 1983		
Software, algorithm	LOOS	Romo and Grossfield, 2009		
Software, algorithm	VMD	Humphrey et al., 1996		

### Cloning, gene expression, and protein purification

The multi-integrating pITy plasmid (Parekh et al., 1996), previously used for overexpression of A_2A_R in *Saccharomyces cerevisiae* (O'Malley et al., 2009), was employed in this study. pITy contains a Gal1–10 promoter for galactose-induced expression, a synthetic pre-pro leader sequence that directs protein trafficking (Clements et al., 1991; Parekh et al., 1995), and the yeast alpha terminator. The genes encoding A_2A_R variants with 10-His C-terminal tag were cloned into pITy downstream of the pre-pro leader sequence using either splice overlapping extension (Bryksin and Matsumura, 2010) or USER cloning using X7 polymerase (Nørholm, 2010; Nour-Eldin et al., 2006). The plasmids were then transformed into *S. cerevisiae* strain BJ5464 (MATα ura3-52 trp1 leu2∆1 his3∆200 pep4::HIS3 prb1∆1.6R can1 GAL) (provided by the lab of Anne Robinson at Carnegie Mellon University) using the lithium-acetate/PEG method (Gietz, 2014). Transformants were selected on YPD G-418 plates (1% yeast extract, 2% peptone, 2% dextrose, 2.0 mg/mL G-418).

Receptor was expressed and purified following the previously described protocol (Niebauer and Robinson, 2006). In brief, from freshly streaked YPD plates (1% yeast extract, 2% peptone, 2% dextrose), single colonies were grown in 5 mL YPD cultures overnight at 30°C. From these 5 mL cultures, 50 mL cultures were grown with a starting OD of 0.5 overnight at 30°C. To induce expression, yeast cells from these 50 mL cultures were centrifuged at 3000 × *g* to remove YPD before resuspended in YPG medium (1% yeast, 2% peptone, 2% D-galactose) at a starting OD of 0.5. The receptor was expressed for 24 hr overnight at 30°C with 250 rpm shaking. Cells were pelleted by centrifugation at 3000 × *g*, washed in sterile PBS buffer, and pelleted again before storage at –80°C until purification.

Mechanical bead lysis of cells was done, per 250 mL of cell culture, by performing 12 pulses of 60 s intense vortexing (with at least 60 s of rest in between pulses) in 10 mL 0.5 mm zirconia silica beads (BioSpec, Bartlesville, OK, USA; #11079105z), 25 mL of lysis buffer (50 mM sodium phosphate, 300 mM sodium chloride, 10% [v/v] glycerol, pH = 8.0, 2% [w/v] n-dodecyl-β-D-maltopyranoside [DDM; Anatrace, Maumee, OH, USA; #D310], 1% [w/v] 3-[(3-cholamidopropyl)dimethylammonio]−1-propanesulfonate [CHAPS; Anatrace; #C216], and 0.2% [w/v] cholesteryl hemisuccinate [CHS; Anatrace; #CH210] and an appropriate amount of 100× Pierce Halt EDTA-free protease inhibitor [Pierce, Rockford, IL, USA; #78439]). Beads were separated using a Kontex column. Unlysed cells were removed by centrifugation at 3220 × *g* for 10 min. Receptor was let solubilized on rotary mixer for 3 hr before cell debris was removed by centrifugation at 10,000 × *g* for 30 min. Solubilized protein was incubated with Ni-NTA resin (Pierce; #88221) overnight. Protein-resin mixture was then washed extensively in purification buffer (50 mM sodium phosphate, 300 mM sodium chloride, 10% [v/v] glycerol, 0.1% [w/v] DDM, 0.1% [w/v] CHAPS and 0.02% [w/v] CHS, pH = 8.0) containing low imidazole concentrations (20–50 mM). A_2A_R was eluted into purification buffer containing 500 mM imidazole. Prior to further chromatographic purification, imidazole was removed using a PD-10 desalting column (GE Healthcare, Pittsburgh, PA, USA; #17085101).

Ligand affinity resin was prepared as previously described for purification of active A_2A_R (O'Malley et al., 2007; Weiß and Grisshammer, 2002). In brief, 8 mL of isopropanol-washed Affigel 10 resin (BioRad, Hercules, CA, USA; #1536099) was mixed gently in an Erlenmeyer flask for 20 hr at room temperature with 48 mL of DMSO containing 24 mg of xanthine amine congener (XAC, high-affinity A_2A_R antagonist, K_D_ = 32 nM; Sigma, St. Louis, MO, USA; #X103). The absorbance at 310 nm of the XAC-DMSO solution before and after the coupling reaction was measured in 10 mM HCl and compared to a standard curve. The amount of resin bound to ligand was estimated to be 5.6 μM. The coupling reaction was quenched by washing the resin with DMSO, then with Tris-HCl 50 mM (pH = 7.4), then with 20% (v/v) ethanol. The resin was packed into a Tricorn 10/50 column (GE Healthcare) under pressure via a BioRad Duoflow FPLC (BioRad).

For purification of active A_2A_R, the column was equilibrated with 4 CV of purification buffer. The IMAC-purified A_2A_R was desalted and diluted to 5.5 mL before applied to a 5 mL sample loop on the BioRad Duoflow FPLC, from which the sample was loaded onto the column at a rate of 0.1 mL/min. Inactive A_2A_R was washed from the column by flowing 10 mL of purification buffer at 0.2 mL/min, followed by 16 mL at 0.4 mL/min. Active A_2A_R was eluted from the column by flowing purification buffer containing 20 mM theophylline (low-affinity A_2A_R antagonist, K_D_ = 1.6 μM; Sigma; #T1633). Western blot analysis was performed to determine 4 mL fractions with active A_2A_R collected with a BioFrac fraction collector (BioRad), which were then concentrated through a 30 kDa MWCO centrifugal filter (Millipore, Billerica, MA, USA; #UFC803096) and desalted to remove excess theophylline. For the experiments where the salt concentrations were varied, the buffer exchange was done also by this last desalting step.

### Size-exclusion chromatography

To separate oligomeric species of active A_2A_R, a prepacked Tricorn Superdex 200 10/300 GL column (GE Healthcare; #17-5175-01) connected to a BioRad Duoflow FPLC was equilibrated with 60 mL of running buffer (150 mM sodium chloride except for the ionic strength experiments where NaCl concentration is adjusted to achieve the desired ionic strengths, 50 mM sodium phosphate, 10% [v/v] glycerol, 0.1% [w/v] DDM, 0.1% [w/v] CHAPS, 0.02% [w/v] CHS, pH = 8.0) at a flow rate of 0.2 mL/min. 0.5 mL fractions were collected with a BioFrac fraction collector in 30 mL of running buffer at the same flow rate. The subsequent SEC analysis performed on the SEC-separated oligomeric populations also followed this protocol.

### SEC peak analysis

SEC chromatograms were analyzed using OriginLab using the nonlinear curve fit (Gaussian) function. The area under the curve and the peak width were manually defined in cases where the SNR of the SEC trace were too low. The R^2^ values reached > 0.96 for most cases. The population of each oligomeric species was expressed as the integral of each Gaussian this curve fit of the SEC signal. The HMW oligomer peak in some cases could not be fitted with one curve and thus was fitted with two curves instead. The reported standard errors were calculated from the variance of the fit and did not correspond to experimental errors. The results are detailed in Figure 1—figure supplement 2 and Supplementary file 1.

### SDS-PAGE and western blotting

10% SDS-PAGE gels were hand-casted in BioRad Criterion empty cassettes (BioRad; #3459902, 3459903). Lysate controls were prepared by lysis of 5 OD cell pellets with 35 μL of YPER (Fisher Scientific, Waltham, MA, USA; #8990) at RT for 20 min, incubation with 2× Laemmli buffer (4% [w/v] SDS, 16% [v/v] glycerol, 0.02% [w/v] bromophenol blue, 167 M Tris, pH 6.8) at 37°C for 1 hr, and centrifugation at 3000 × *g* for 1 min to pellet cell debris. Protein samples were prepared by incubation with 2× Laemmli buffer at 37°C for 30 min. For all samples, 14 μL (for 26-well gel) or 20 μL (for 18-well gel) was loaded per lane, except for 7 μL of Magic Mark XP Western protein ladder (Thermo Scientific, Waltham, MA, USA; #LC5602) as a standard. Electrophoresis was carried out at 120 V for 100 min. Proteins were transferred to 0.2 μm nitrocellulose membranes (BioRad; #170-4159) via electroblotting using a BioRad Transblot Turbo, mixed MW protocol. Membranes were blocked in Tris-buffered saline with Tween (TBST; 150 mM sodium chloride, 15.2 mM Tris-HCl, 4.6 mM Tris base, pH = 7.4, 0.1% [v/v] Tween 20 [BioRad; #1706531]) containing 5% (w/v) dry milk, then probed with anti-A_2A_R antibody, clone 7F6-G5-A2, mouse monoclonal (Millipore, Burlington, MA, USA; #05-717) at 1:500 in TBST with 0.5% (w/v) dry milk. Probing with secondary antibody was done with a fluorescent anti-mouse IgG H&L DyLight 550 antibody (Abcam, Cambridge, MA, USA; #ab96880) at 1:600 in TBST containing 0.5% (w/v) milk.

Western blot was analyzed with Image Lab 6.1 software (BioRad), with built-in tool to define each sample lane and to generate an intensity profile. Peaks were manually selected and integrated with the measure tool to determine the amount of protein present.

### CGMD simulations

Initial configuration of A_2A_R was based on the crystal structure of the receptor in the active state (PDB 5G53). Since this structure does not include the entire C-terminus, we resorted to using homology modeling software (i.e., MODELLER 9.23) (Eswar et al., 2006) to predict the structures of the C-terminus. After removing all non-receptor components, the first segment of the C-terminus consisting of residues 291–314 was modeled as a helical segment parallel to the cytoplasmic membrane surface while the rest of the C-terminus was modeled as intrinsically disordered. MODELLER is much more accurate in structural predictions for segments less than 20 residues. This limitation necessitated that we run an equilibrium MD simulation for 2 µs to obtain a well-equilibrated structure that possesses a more viable starting conformation. To validate our models of all potential variants of A_2A_R, we calculated the RMSD and RMSF for each respective system. Default protonation states of ionizable residues were used. The resulting structure was converted to MARTINI CG topology using the martinize.py script (de Jong et al., 2013). The ELNeDyn elastic network (Periole et al., 2009) was used to constrain protein secondary and tertiary structures with a force constant of 500 kJ/mol/nm^2^ and a cutoff of 1.5 nm. To optimize loop refinement of the model, a single copy was embedded in a 1-palmitoyl-2-oleoyl-sn-glycero-3-phosphocholine (POPC) bilayer using the insane.py script, solvated with MARTINI polarizable water, neutralized with 0.15 M NaCl, and a short MD (1.5 µs) run to equilibrate the loop regions. Subsequently, two monomers of the equilibrated A_2A_R were randomly rotated and placed at the center of a 13 nm × 13 nm × 11 nm (xyz) box, 3.5 nm apart, with their principal transmembrane axis aligned parallel to the z axis. The proteins were then embedded in a POPC bilayer using the insane.py script. Sodium and chloride ions were added to neutralize the system and obtain a concentration of 0.15 M NaCl. Total system size was typically in the range of 34,000 CG particles, with a 280:1 lipid:protein ratio. Ten independent copies were generated for each A_2A_R truncated variant. v2.2 of the MARTINI CG force field (Monticelli et al., 2008) was used for the protein and water, and v2.0 was used for POPC. All CG simulations were carried out in GROMACS 2016 (Abraham et al., 2015) in the NPT ensemble (P = 1 atm, T = 310 K). The Bussi velocity rescaling thermostat was used for temperature control with a coupling constant of τ_t_ = 1.0 ps (Bussi et al., 2007), while the Parrinello–Rahman barostat (Martonák et al., 2003) was used to control the pressure semi-isotropically with a coupling constant of τ_t_ = 12.0 ps and compressibility of 3 × 10^–4^ bar^–1^. Reaction field electrostatics was used with Coulomb cutoff of 1.1 nm. Non-bonded Lennard–Jones interactions were treated with a cutoff of 1.1 nm. All simulations were run with a 15 fs time step, updating neighbor lists every 10 steps. Cubic periodic boundary conditions along the x, y, and z axes were used. Each simulation was run for 8 µs.

### Atomistic MD simulations

Three snapshots of symmetric dimers of A_2A_R for each respective truncated variant were randomly selected from the CG simulations as starting structures for backmapping. CG systems were converted to atomistic resolution using the backward.py script (Wassenaar et al., 2014). All simulations were run in Gromacs2019 in the *NPT* ensemble (P = 1 bar, T = 310 K) with all bonds restrained using the LINCS method (Hess et al., 1997). The Parrinello–Rahman barostat was used to control the pressure semi-isotropically with a coupling constant of τ_t_ = 1.0 ps and a compressibility of 4.5 × 10^–5^ bar^–1^, while the Bussi velocity rescaling thermostat was used for temperature control with a coupling constant of τ_t_ = 0.1 ps. Proteins, lipids, and solvents were separately coupled to the thermostat. The CHARMM36 and TIP3P force fields (Best et al., 2012; Jorgensen et al., 1983) were used to model all molecular interactions. Periodic boundary conditions were set in the x, y, and z directions. Particle mesh Ewald (PME) electrostatics was used with a cutoff of 1.0 nm. A 2-fs time step was used for all atomistic runs, and each simulation was run for 50 ns.

### Analysis of computational results

All trajectories were postprocessed using gromacs tools and in-house scripts. We ran a clustering analysis of all dimer frames from the CG simulations using Daura et al.’s clustering algorithm (Daura et al., 1999) implemented in GROMACS, with an RMSD cutoff of 1.5 Å. An interface was considered dimeric if the minimum center of mass distance between the protomers was less than 5 Å. This method uses an RMSD cutoff to group all conformations with the largest number of neighbors into a cluster and eliminates these from the pool, then repeats the process until the pool is empty. We focused our analysis on the most populated cluster from each truncated variant. Electrostatic interactions in the dimer were calculated from CG systems with LOOS (Romo and Grossfield, 2009) using a distance cutoff of 5.0 Å. Transmembrane helical tilt angles were also calculated in LOOS from CG simulations. Hydrogen bonds were calculated from AA simulations using the hydrogen bonds plugin in VMD (Humphrey et al., 1996), with a distance cutoff of 3.5 Å and an angle cutoff of 20°. Only C-terminal residues were included in hydrogen bond analysis. PyMOL (The PyMOL Molecular Graphics System, version 2.0, Schrödinger, LLC, 2020) was used for molecular visualizations.

### Assessing A_2A_R oligomerization with increasing ionic strength

Na_2_HPO_4_ and NaH_2_PO_4_ in the buffer make up an ionic strength of 0.15 M, to which NaCl was added to increase the ionic strength to 0.45 M and furthermore to 0.95 M. The A_2A_R variants were purified at 0.45 M ionic strength and then exchanged into buffers of different ionic strengths using a PD-10 desalting column prior to subjecting the samples to SEC. The buffer composition is detailed below.

**Table:** 

Buffers	Components	Concentration (mM)	Ionic strength (mM)
0.15 M ionic strength	NaCl	0	0
	NaH_2_PO_4_	4	4
	Na_2_HPO_4_	49	146
0.45 M ionic strength	NaCl	300	300
	NaH_2_PO_4_	4	4
	Na_2_HPO_4_	49	146
0.95 M ionic strength	NaCl	800	800
	NaH_2_PO_4_	4	4
	Na_2_HPO_4_	49	146

### Isolated C-terminus purification

*Escherichia coli* BL21 (DE3) cells (Sigma; #CMC0014) were transfected with pET28a DNA plasmids containing the desired A_2A_R sequence with a 6x His tag attached for purification. Cells from glycerol stock were grown in 10 mL luria broth (LB, Sigma Aldrich, L3022) overnight at 37°C and then used to inoculate 1 L of fresh LB and 10 μg/mL kanamycin (Fisher Scientific, BP906). Growth of cells was performed at 37°C, 200 rpm until optical density at λ = 600 nm reached 0.6–0.8. Expression was induced by incubation with 1 mM isopropyl-β-D-thiogalactoside (Fisher Bioreagents, BP175510) for 3 hr.

Cells were harvested with centrifugation at 5000 rpm for 30 min. Harvested cells were resuspended in 25 mL Tris-HCl, pH = 7.4, 100 mM NaCl, 0.5 mM DTT, 0.1 mM EDTA with 1 Pierce protease inhibitor tablet (Thermo Scientific, A32965), 1 mM PMSF, 2 mg/mL lysozyme, 20 μg/mL DNase (Sigma, DN25) and 10 mM MgCl_2_, and incubated on ice for 30 min. Samples were then incubated at 30°C for 20 min, then flash frozen and thawed three times in LN_2_. Samples were then centrifuged at 10,000 rpm for 10 min to remove cell debris. 1 mM PMSF was added again and the resulting supernatant was incubated while rotating for at least 4 hr with Ni-NTA resin. The resin was loaded to a column and washed with 25 mL 20 mM sodium phosphate, pH = 7.0, 1 M NaCl, 20 mM imidazole, 0.5 mM DTT, 100 μM EDTA. Purified protein was eluted with 15 mL of 20 mM sodium phosphate, pH = 7.0, 0.5 mM DTT, 100 mM NaCl, 300 mM imidazole. The protein was concentrated to a volume of 2.5 mL and was buffer exchanged into 20 mM ammonium acetate buffer, pH = 7.4, 100 mM NaCl using a GE PD-10 desalting column. Purity of sample was confirmed with SDS-PAGE and western blot.

### Aggregation assay to assess A_2A_R C-terminus assembly

Absorbance was measured at 450 nm using a Shimadzu UV-1601 spectrophotometer with 120 µL sample size. Prior to reading, samples were incubated at 40°C for 5 min. Samples were vigorously pipetted to homogenize any precipitate before absorbance was measured. Protein concentration was 50 µM in a 20 mM ammonium acetate buffer (pH = 7.4).

### Differential scanning fluorimetry (DSF)

DSF was conducted with a BioRad CFX90 real-time PCR machine. A starting temperature of 20°C was increased at a rate of 0.5°C per 30 s to a final temperature of 85°C. All samples contained 40 μL of 40 µM A_2A_R C-terminus, 9x SYPRO orange (ThermoFisher S6650), 200 mM NaCl, and 20 mM MES. Fluorescence was detected in real time at 570 nm. All samples were conducted in triplicate.

### Hydrophobicity and charge profile of C-terminus

The hydrophobicity profile reported in Figure 6—figure supplement 1 was determined with ProtScale using method described by Kyte and Doolittle, 1982, window size of 3.

## Data Availability

All data generated or analysed during this study are included in the manuscript and supporting files.

## Additional files

Supplementary file 1. Results from curve fitting using OriginLab and calculations of the high-molecular-weight (HMW) oligomer and dimer levels for all A_2A_R variants used in the main text of this study. The variants are grouped by the order they appear and numbered corresponding to Figure 1—figure supplement 2. The levels of dimer and HMW oligomer are expressed relative to the monomeric population in arbitrary units as monomer-equivalent concentration ratios. The errors are calculated from the variance of the fit, not experimental variations, and are within 95% confidence interval. Only the WT replicates are represented with standard deviation as experimental variations (last row; n = 5; mean ± SD).

Transparent reporting form
