# Supplementary figures and images for: Homo-oligomerization of the human adenosine A2A receptor is driven by the intrinsically disordered C-terminus
Source: eLife. 2021 Jul 16;10:e66662. doi: 10.7554/eLife.66662 (PMC8328514; doi:10.7554/eLife.66662)

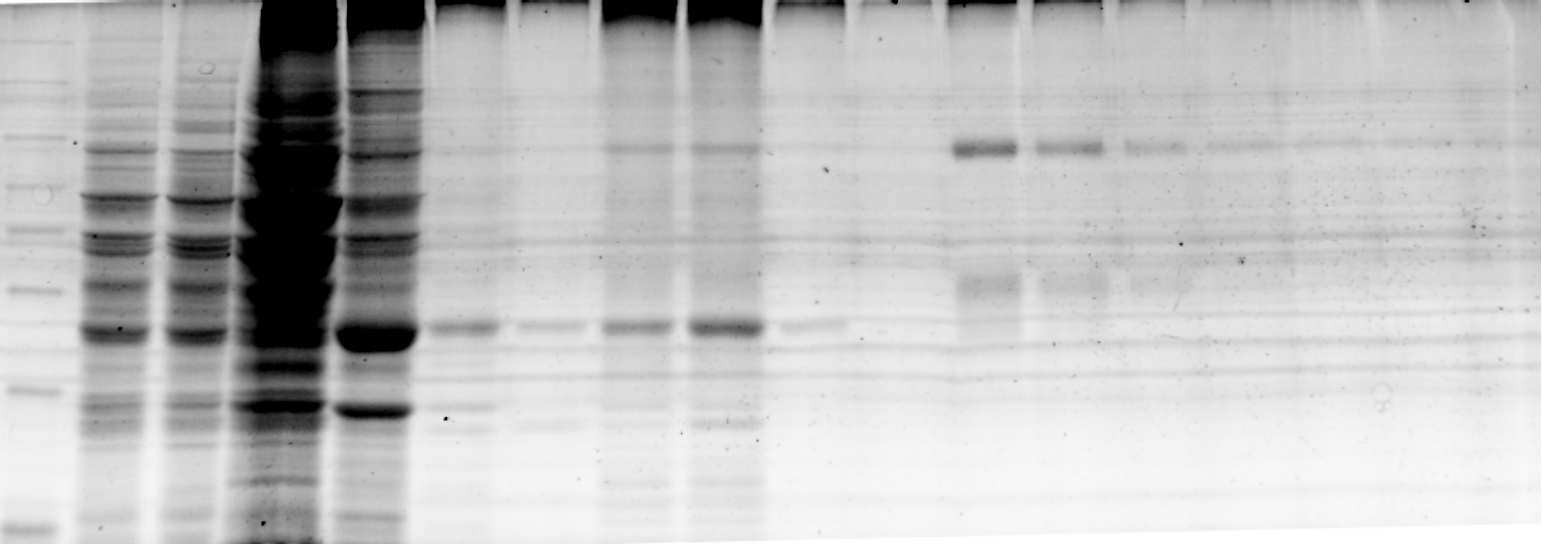

Supplement: Figure 1—figure supplement 1—source data 1. [file elife-66662-fig1-figsupp1-data1.tif.zip › Figure1-figuresupplement1-sourcedata1.tif]

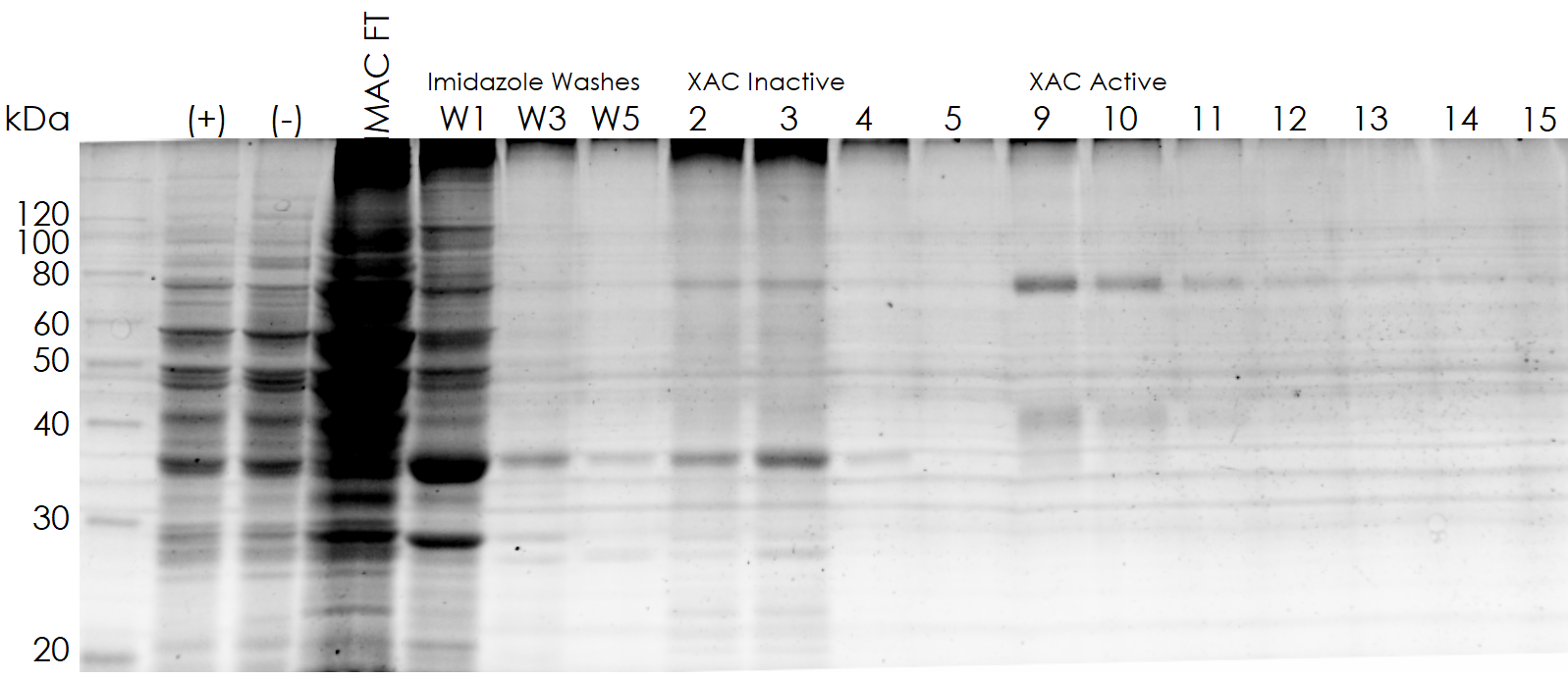

Supplement: Figure 1—figure supplement 1—source data 2. — Positive ([+] ctrl) and negative ([–] ctrl) controls consist of 5 OD cell lysate of Saccharomyces cerevisiae BJ5464 cells expressing and not expressing A2AR WT, respectively. ‘IMAC FT’ indicates the flow-through from IMAC step. ‘XAC inactive’ and ‘XAC active’ indicate the fractions that do not and do bind to XAC during the ligand-affinity chromatography step. MagicMark protein ladder (LC5602) is used as the molecular weight standard. [file elife-66662-fig1-figsupp1-data2.tif.zip › Figure 1-figure supplement 1-source data 2.tif]

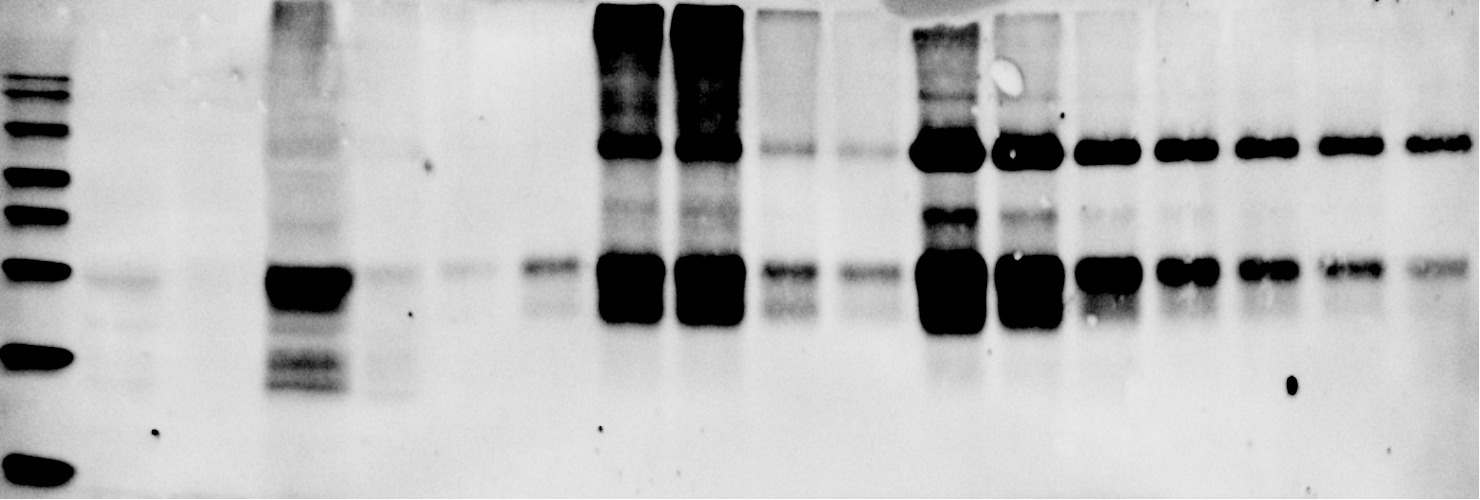

Supplement: Figure 1—figure supplement 1—source data 3. [file elife-66662-fig1-figsupp1-data3.tif.zip › Figure 1-figure supplement 1-source data 3.tif]

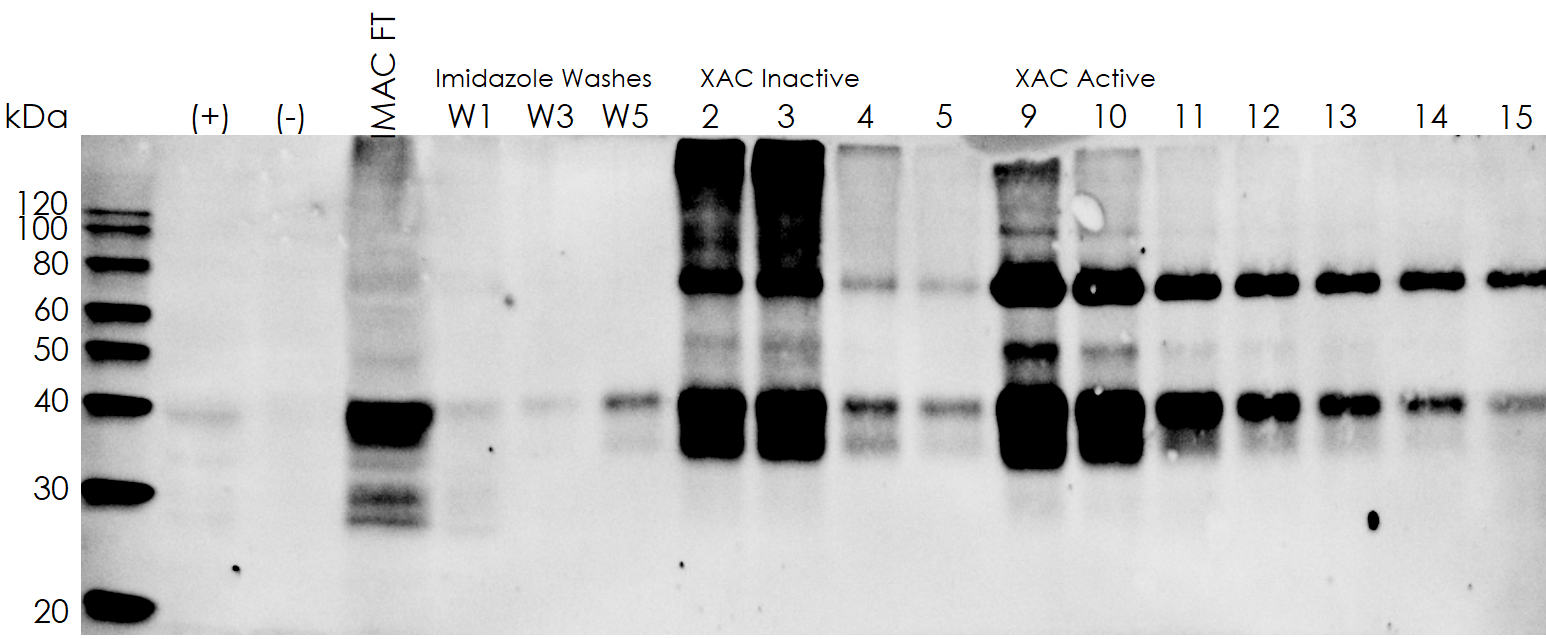

Supplement: Figure 1—figure supplement 1—source data 4. — Positive ([+] ctrl) and negative ([–] ctrl) controls consist of 5 OD cell lysate of Saccharomyces cerevisiae BJ5464 cells expressing and not expressing A2AR WT, respectively. ‘IMAC FT’ indicates the flow-through from IMAC step. ‘XAC inactive’ and ‘XAC active’ indicate the fractions that do not and do bind to XAC during the ligand-affinity chromatography step. MagicMark protein ladder (LC5602) is used as the molecular weight standard. [file elife-66662-fig1-figsupp1-data4.tif.zip › Figure 1-figure supplement 1-source data 4.tif]

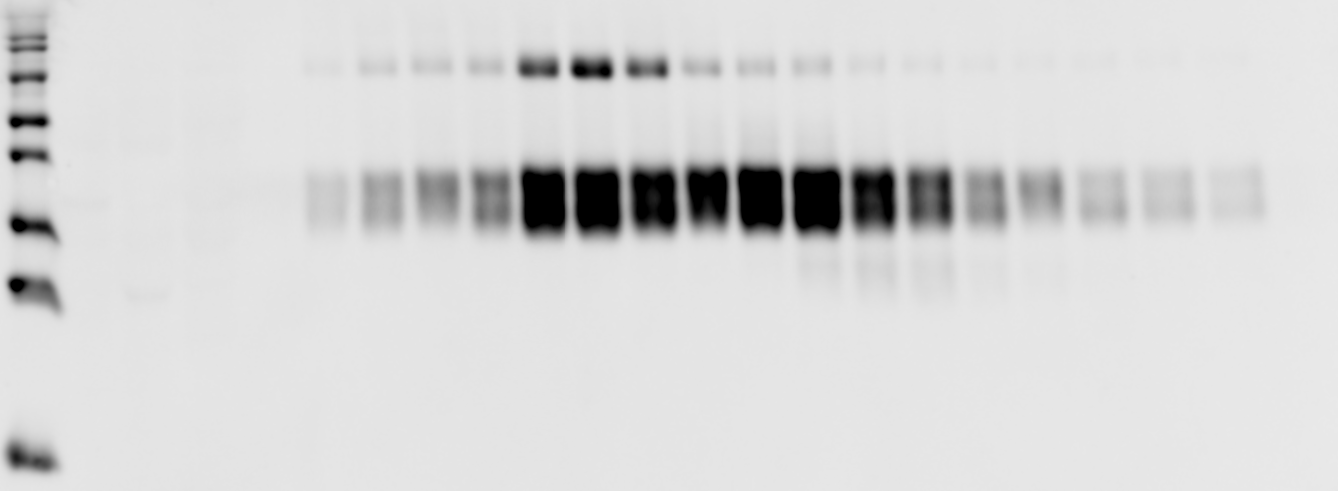

Supplement: Figure 1—figure supplement 1—source data 5. [file elife-66662-fig1-figsupp1-data5.tif.zip › Figure 1-figure supplement 1-source data 5.tif]

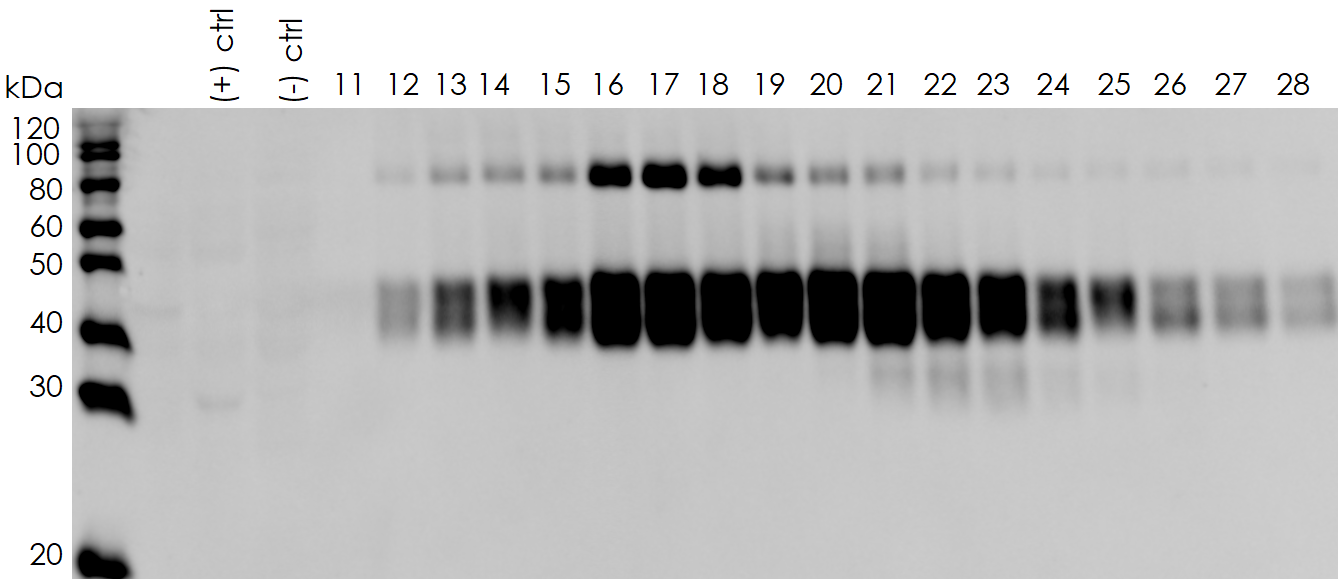

Supplement: Figure 1—figure supplement 1—source data 6. — Each lane on the blot is from 0.5 mL fractions eluted from a Superdex 200 10/300 GL (GE Healthcare) column. MagicMark protein ladder (LC5602) is used as the molecular weight standard. [file elife-66662-fig1-figsupp1-data6.tif.zip › Figure 1-figure supplement 1-source data 6.tif]

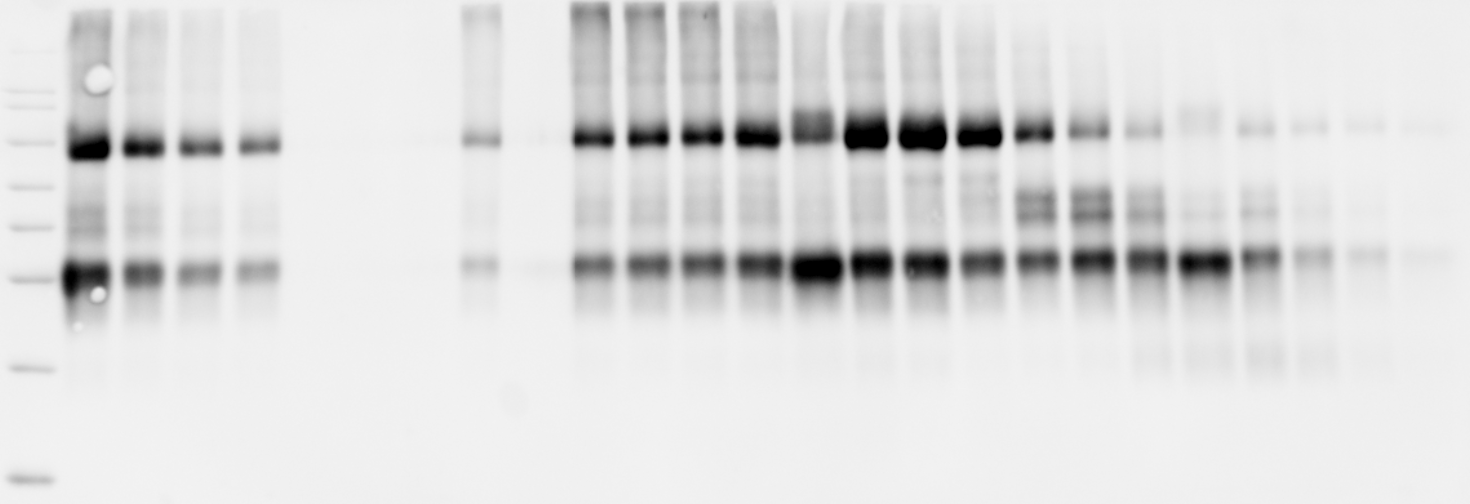

Supplement: Figure 2—source data 1. [file elife-66662-fig2-data1.tif.zip › Figure 2-source data 1.tif]

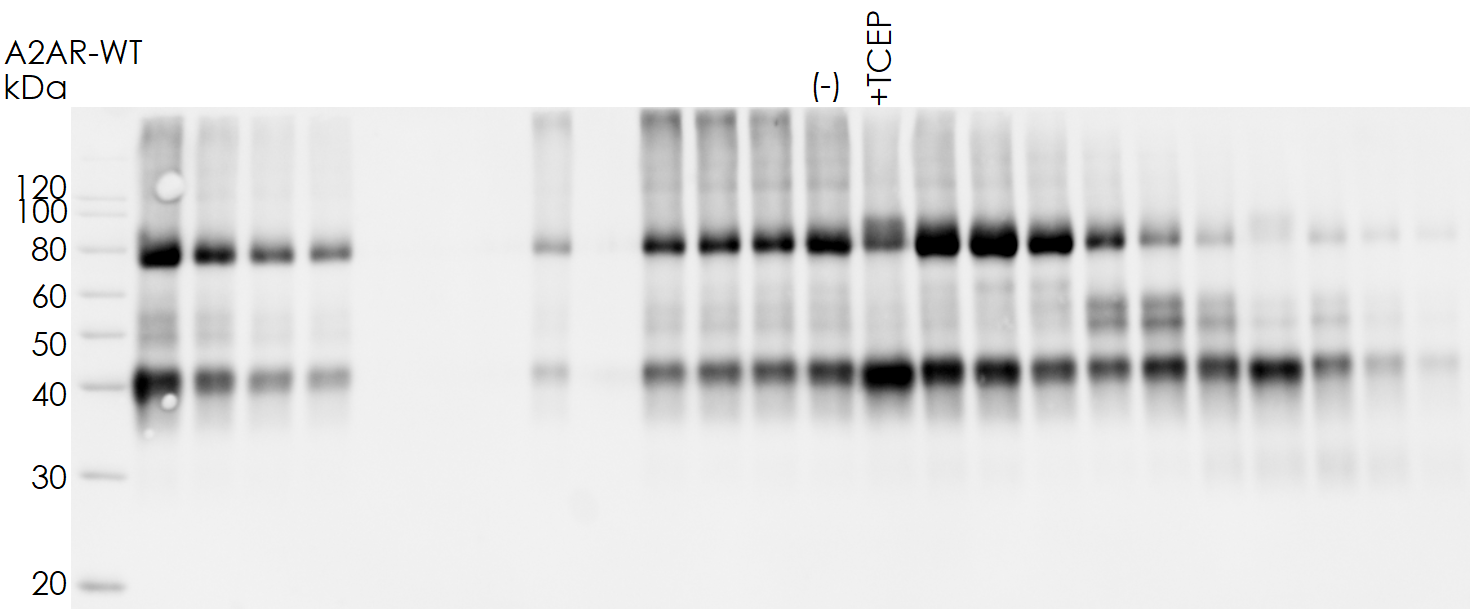

Supplement: Figure 2—source data 2. — MagicMark protein ladder (LC5602) is used as the molecular weight standard. [file elife-66662-fig2-data2.tif.zip › Figure 2-source data 2.tif]

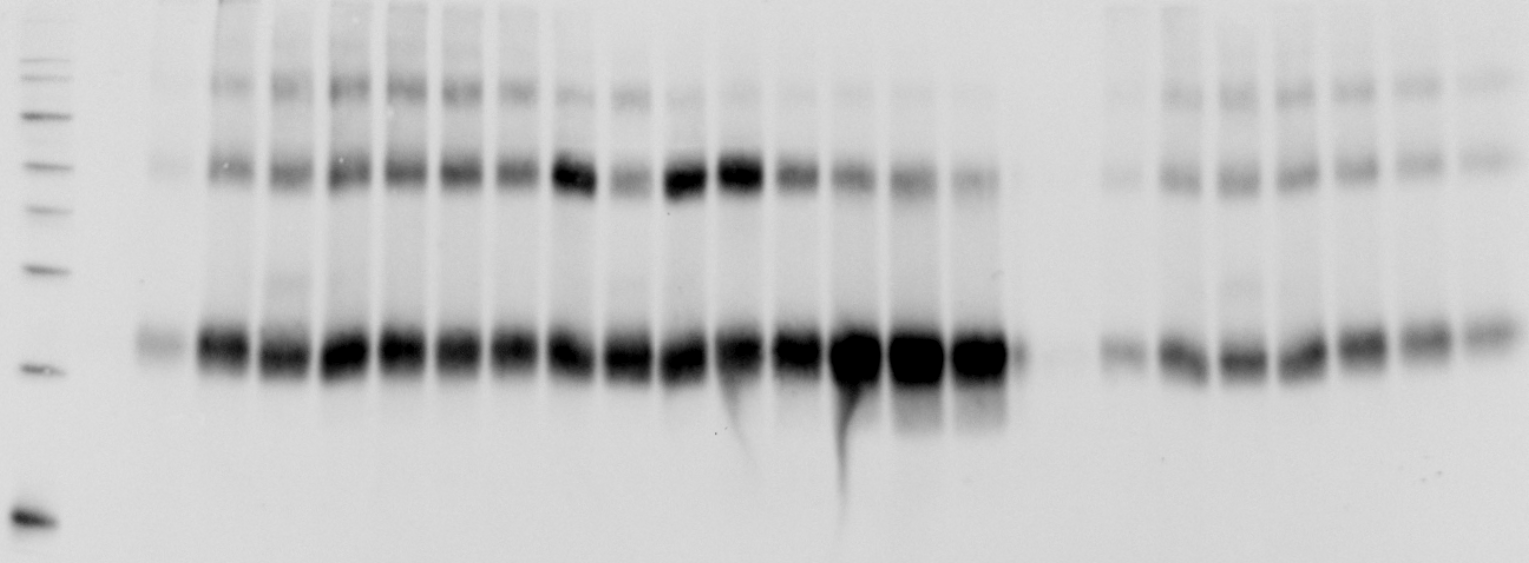

Supplement: Figure 2—source data 3. [file elife-66662-fig2-data3.tif.zip › Figure 2-source data 3.tif]

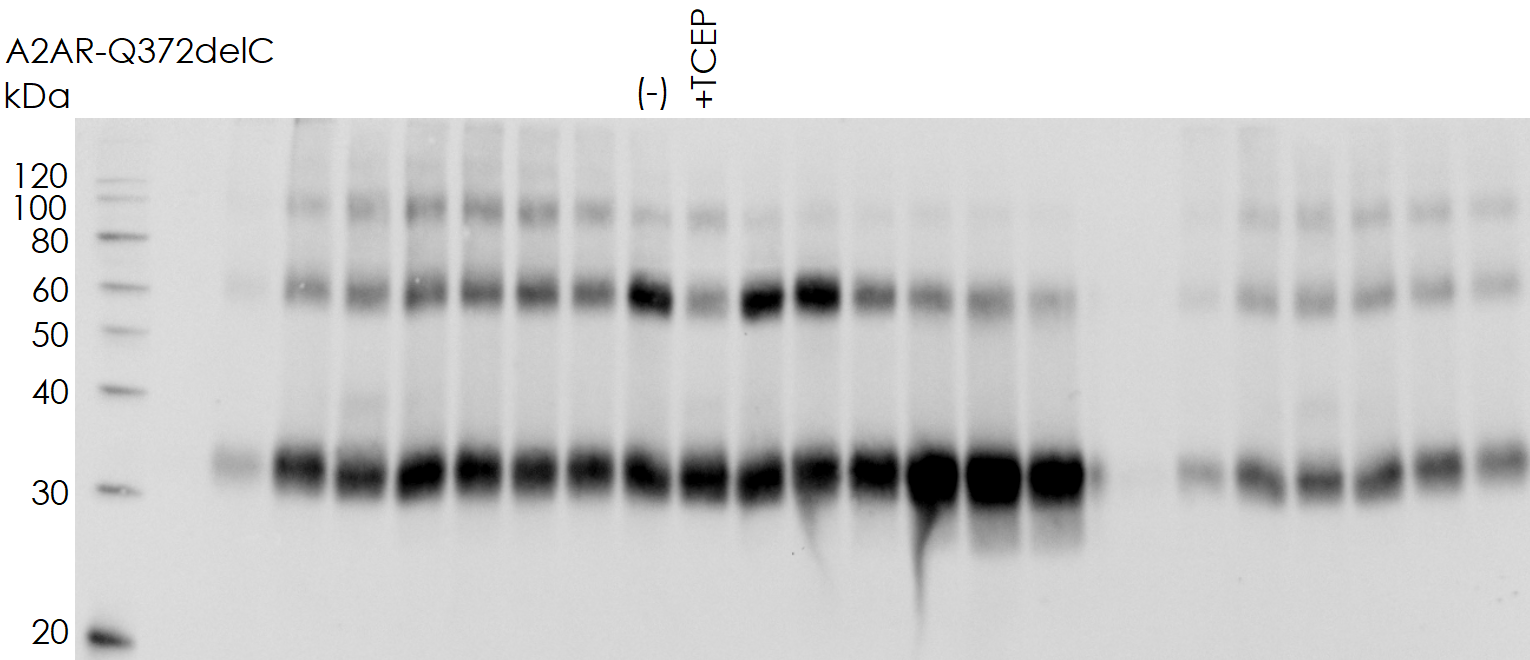

Supplement: Figure 2—source data 4. — MagicMark protein ladder (LC5602) is used as the molecular weight standard. [file elife-66662-fig2-data4.tif.zip › Figure 2-source data 4.tif]
