# Supplementary material for: Homo-oligomerization of the human adenosine A2A receptor is driven by the intrinsically disordered C-terminus
Source: eLife. 2021 Jul 16;10:e66662. doi: 10.7554/eLife.66662 (PMC8328514; doi:10.7554/eLife.66662)
Supplement: Supplementary file 1. — The variants are grouped by the order they appear and numbered corresponding to Figure 1—figure supplement 2. The levels of dimer and HMW oligomer are expressed relative to the monomeric population in arbitrary units as monomer-equivalent concentration ratios. The errors are calculated from the variance of the fit, not experimental variations, and are within 95% confidence interval. Only the WT replicates are represented with standard deviation as experimental variations (last row; n = 5; mean ± SD). [file elife-66662-supp1.docx]

| **Fig** | **Variants** | **No.** | **HMW Oligomer Level** | **Dimer Level** | **Total Oligomer Level** | **[HMW Oligomer]** | **[Dimer]** | **[Monomer]** |
| --- | --- | --- | --- | --- | --- | --- | --- | --- |
| 2A | WT | 1 | 0.20 ± 0.01 | 1.14 ± 0.01 | 1.34 ± 0.01 | 1.77 ± 0.05 | 10.39 ± 0.05 | 9.09 ± 0.07 |
|  | C394S | 2 | 0.28 ± 0.06 | 0.57 ± 0.01 | 0.85 ± 0.06 | 1.66 ± 0.35 | 3.36 ± 0.07 | 5.90 ± 0.06 |
|  | C394A | 3 | 0.31 ± 0.08 | 0.28 ± 0.06 | 0.59 ± 0.10 | 0.49 ± 0.11 | 0.44 ± 0.10 | 1.57 ± 0.08 |
|  | C394L | 4 | 0.78 ± 0.01 | 0.43 ± 0.01 | 1.21 ± 0.01 | 9.09 ± 0.13 | 5.07 ± 0.07 | 11.73 ± 0.09 |
|  | C394M | 5 | 0.50 ± 0.08 | 0.38 ± 0.03 | 0.88 ± 0.09 | 2.70 ± 0.42 | 2.05 ± 0.18 | 5.44 ± 0.05 |
|  | C394V | 6 | 0.64 ± 0.01 | 0.23 ± 0.01 | 0.88 ± 0.01 | 9.94 ± 0.13 | 3.65 ± 0.06 | 15.44 ± 0.07 |
| 3B | WT | 1 | 0.20 ± 0.01 | 1.14 ± 0.01 | 1.34 ± 0.01 | 1.77 ± 0.05 | 10.39 ± 0.05 | 9.09 ± 0.07 |
|  | P395ΔC | 7 | 0.58 ± 0.01 | 1.15 ± 0.01 | 1.73 ± 0.02 | 3.34 ± 0.05 | 6.69 ± 0.05 | 5.80 ± 0.06 |
|  | Q372ΔC | 8 | 0.22 ± 0.01 | 0.65 ± 0.01 | 0.87 ± 0.01 | 1.64 ± 0.05 | 4.95 ± 0.05 | 7.59 ± 0.06 |
|  | N359ΔC | 9 | 0.28 ± 0.01 | 0.81 ± 0.01 | 1.09 ± 0.01 | 2.31 ± 0.06 | 6.72 ± 0.05 | 8.30 ± 0.06 |
|  | P354ΔC | 10 | 0.42 ± 0.01 | 0.19 ± 0.01 | 0.62 ± 0.02 | 2.17 ± 0.05 | 0.99 ± 0.05 | 5.12 ± 0.05 |
|  | G349ΔC | 11 | 0.48 ± 0.02 | 0.09 ± 0.01 | 0.58 ± 0.02 | 2.23 ± 0.07 | 0.42 ± 0.06 | 4.60 ± 0.03 |
|  | G344ΔC | 12 | 0.44 ± 0.10 | 0.06 ± 0.06 | 0.50 ± 0.12 | 0.80 ± 0.18 | 0.11 ± 0.11 | 1.81 ± 0.04 |
|  | V334ΔC | 13 | 0.04 ± 0.01 | 0.10 ± 0.01 | 0.14 ± 0.01 | 0.29 ± 0.06 | 0.83 ± 0.06 | 8.23 ± 0.06 |
|  | A316ΔC | 14 | 0.03 ± 0.01 | 0.03 ± 0.01 | 0.06 ± 0.01 | 0.08 ± 0.02 | 0.08 ± 0.02 | 2.89 ± 0.02 |
| 3C | WT | 15 | 0.88 ± 0.04 | 0.49 ± 0.01 | 1.37 ± 0.01 | 5.37 ± 0.22 | 2.98 ± 0.07 | 6.10 ± 0.04 |
|  | WT-ERRAAA | 16 | 0.66 ± 0.03 | 0.29 ± 0.01 | 0.95 ± 0.03 | 3.76 ± 0.16 | 1.64 ± 0.08 | 5.72 ± 0.07 |
|  | N359ΔC | 17 | 0.68 ± 0.04 | 0.33 ± 0.03 | 1.01 ± 0.05 | 1.10 ± 0.06 | 0.53 ± 0.04 | 1.61 ± 0.04 |
|  | N359ΔC-ERRAAA | 18 | 0.38 ± 0.03 | 0.48 ± 0.02 | 0.85 ± 0.04 | 1.05 ± 0.08 | 1.32 ± 0.06 | 2.78 ± 0.05 |
| 4 | WT 0.15 M | 19 | 0.07 ± 0.01 | 0.09 ± 0.01 | 0.16 ± 0.02 | 0.19 ± 0.04 | 0.27 ± 0.04 | 2.87 ± 0.04 |
|  | WT 0.45 M | 15 | 0.88 ± 0.04 | 0.49 ± 0.01 | 1.37 ± 0.04 | 5.37 ± 0.22 | 2.98 ± 0.07 | 6.10 ± 0.04 |
|  | WT 0.95 M | 20 | 2.20 ± 0.04 | 1.31 ± 0.02 | 3.51 ± 0.05 | 14.54 ± 0.25 | 8.62 ± 0.11 | 6.60 ± 0.06 |
|  | WT-ERRAAA 0.15 M | 21 | 0.17 ± 0.05 | 0.02 ± 0.01 | 0.19 ± 0.05 | 0.62 ± 0.17 | 0.07 ± 0.01 | 3.73 ± 0.03 |
|  | WT-ERRAAA 0.45 M | 16 | 0.47 ± 0.08 | 0.45 ± 0.04 | 0.92 ± 0.09 | 2.55 ± 0.45 | 2.45 ± 0.23 | 5.45 ± 0.07 |
|  | WT-ERRAAA 0.95 M | 22 | 1.20 ± 0.03 | 0.38 ± 0.01 | 1.58 ± 0.03 | 7.41 ± 0.18 | 2.37 ± 0.08 | 6.21 ± 0.04 |
|  | N359ΔC 0.15 M | 23 | 0.11 ± 0.01 | 0.11 ± 0.01 | 0.21 ± 0.02 | 0.72 ± 0.08 | 0.71 ± 0.08 | 6.67 ± 0.07 |
|  | N359ΔC 0.45 M | 17 | 0.68 ± 0.04 | 0.33 ± 0.03 | 1.01 ± 0.05 | 1.10 ± 0.06 | 0.53 ± 0.04 | 1.61 ± 0.04 |
|  | N359ΔC 0.95 M | 24 | 0.04 ± 0.01 | 0.04 ± 0.01 | 0.09 ± 0.01 | 0.51 ± 0.05 | 0.59 ± 0.05 | 11.90 ± 0.06 |
|  | V334ΔC 0.15 M | 25 | 0.13 ± 0.01 | 0.08 ± 0.01 | 0.21 ± 0.01 | 0.65 ± 0.04 | 0.41 ± 0.03 | 5.03 ± 0.03 |
|  | V334ΔC 0.45 M | 13 | 0.04 ± 0.01 | 0.10 ± 0.01 | 0.14 ± 0.01 | 0.29 ± 0.06 | 0.83 ± 0.06 | 8.23 ± 0.06 |
|  | V334ΔC 0.95 M | 26 | 0.09 ± 0.02 | 0.15 ± 0.04 | 0.23 ± 0.01 | 0.85 ± 0.19 | 1.41 ± 0.34 | 9.68 ± 0.27 |
| WT Replicates  (with Variations from the Fit) | | | 1.16 ± 0.05 | 0.65 ± 0.03 | 1.81 ± 0.06 | 9.45 ± 0.39 | 5.34 ± 0.20 | 8.16 ± 0.04 |
|  |  |  | 0.98 ± 0.03 | 0.57 ± 0.01 | 1.56 ± 0.04 | 6.44 ± 0.20 | 3.76 ± 0.09 | 6.55 ± 0.04 |
|  |  |  | 1.48 ± 0.05 | 0.57 ± 0.01 | 2.05 ± 0.05 | 12.02 ± 0.35 | 4.66 ± 0.06 | 8.12 ± 0.05 |
|  |  |  | 0.20 ± 0.01 | 1.14 ± 0.01 | 1.34 ± 0.01 | 1.77 ± 0.05 | 10.39 ± 0.05 | 9.09 ± 0.07 |
|  |  |  | 0.88 ± 0.04 | 0.49 ± 0.01 | 1.37 ± 0.04 | 5.37 ± 0.22 | 2.98 ± 0.07 | 6.10 ± 0.04 |
| WT Replicates (with Experimental Variations: Mean ± SD; n = 5) | | | 0.94 ± 0.47 | 0.68 ± 0.26 | 1.63 ± 0.30 | 7.01 ± 3.92 | 5.42 ± 2.92 | 7.60 ± 1.24 |
